# Excitation Pulse Duration Response of Upconversion Nanoparticles and Its Applications

**DOI:** 10.1021/acs.jpclett.2c03037

**Published:** 2022-11-29

**Authors:** Lucía Labrador-Páez, Uliana Kostiv, Qingyun Liu, Yuanyuan Li, Hans Ågren, Jerker Widengren, Haichun Liu

**Affiliations:** † Department of Applied Physics, KTH Royal Institute of Technology, SE-10691Stockholm, Sweden; ‡ Department of Theoretical Chemistry and Biology, KTH Royal Institute of Technology, SE-10691Stockholm, Sweden; § Wallenberg Wood Science Center, Department of Fibre and Polymer Technology, KTH Royal Institute of Technology, SE-10691Stockholm, Sweden; ∥ Department of Physics and Astronomy, Uppsala University, UppsalaSE-75120, Sweden

## Abstract

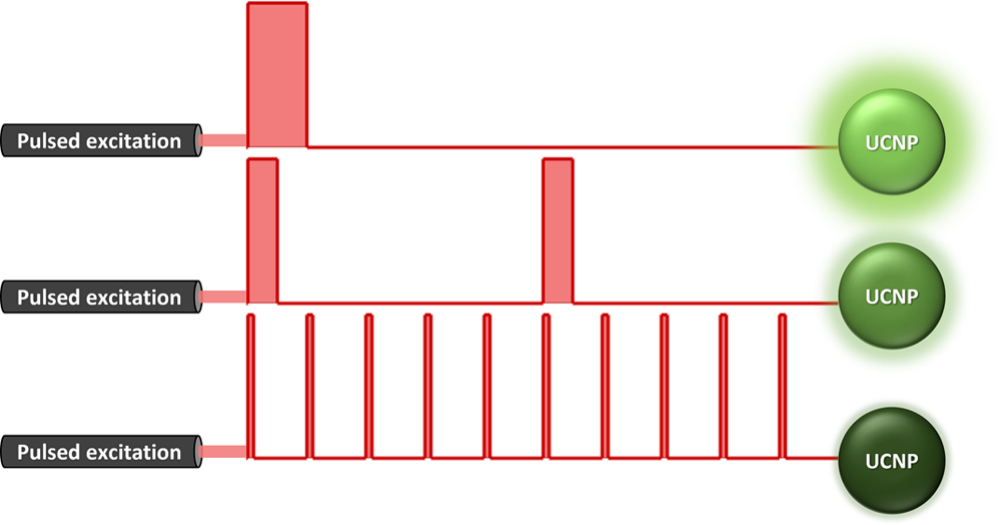

Lanthanide-doped upconversion nanoparticles (UCNPs) have rich photophysics exhibiting complex luminescence kinetics. In this work, we thoroughly investigated the luminescence response of UCNPs to excitation pulse durations. Analyzing this response opens new opportunities in optical encoding/decoding and the assignment of transitions to emission peaks and provides advantages in applications of UCNPs, e.g., for better optical sectioning and improved luminescence nanothermometry. Our work shows that monitoring the UCNP luminescence response to excitation pulse durations (while keeping the duty cycle constant) by recording the average luminescence intensity using a low-time resolution detector such as a spectrometer offers a powerful approach for significantly extending the utility of UCNPs.

Lanthanide-doped upconversion nanoparticles (UCNPs) can convert low-energy near-infrared (NIR) photons into higher-energy NIR, visible, or even ultraviolet photons through subsequent absorption and accumulation of the energy of two or more excitation photons. Lanthanide photon upconversion circumvents the need for high-intensity short-pulse lasers, required in conventional coherent nonlinear optical processes, and thereby provides unique advantages and opportunities for applications. With the advances in upconversion nanochemistry and nanophotonics during the past two decades, UCNPs have been developed as an important group of luminescent nanomaterials with many applications in fields such as nanomedicine,^1−3^ photonics,^4^ and information technology.^5^

UCNPs are composed of a large number of optical centers (lanthanide dopants) accommodated in a host lattice. The mutual interactions between these optical centers following light excitation make each nanoparticle operate as a kinetic optical system, exhibiting fascinating responses to various optical stimuli in the spectral, polarization, intensity, and time domains.^6^ For instance, in the time domain, the luminescence of UCNPs typically exhibits complex kinetics, featuring rise and decay processes encoded with rich information upon multiphotonic excitation with a short pulse.^7,8^

Pulsed excitation is a commonly used approach in the investigation and applications of luminescent materials. As a distinguished example, transient state (TRAST) spectroscopy/microscopy based on pulsed excitation makes it possible to monitor photoinduced, dark state kinetics of fluorescent molecules in a widely applicable manner, by analyzing how the plain, time-averaged fluorescence intensity from organic fluorophores, or even autofluorescent compounds, changes upon systematically varying the modulation of the excitation light.^9,10^ Using the excitation pulse duration response of fluorophores, the TRAST concept has proven to be a powerful tool in cellular and molecular studies, for monitoring sparse molecular interactions,^11^ local oxygenation,^12^ and redox conditions^11^ in cells and solutions, via the dark state transitions of the fluorophores. This information is often difficult to obtain by detecting regular fluorescence parameters, if possible by other methods, before the introduction of the TRAST concept. Various approaches based on pulsed excitation have also proven to be useful for the characterization of UCNPs and for developing their applications. For example, due to the nonlinear excitation rate dependence, quasi-continuous-wave (millisecond pulse duration) excitation of UCNPs has proven to be superior in several applications, such as UCNP-based biomedical imaging^13^ and UCNP optogenetics.^14^ Pulsed excitation has also proven to be useful in applications tuning the emission color of UCNPs^15−17^ and for selectively switching the high-order multiphotonic emission of UCNPs on and off.^18^ However, despite these studies, the pulsed excitation response of UCNPs has not been systematically investigated and its applications have remained underexplored.

In this work, we adapted an excitation pulse duration response procedure from TRAST spectroscopy of organic fluorophores to thoroughly investigate the rich photophysics of Yb^3+^-Er^3+^-codoped and Yb^3+^-Tm^3+^-codoped UCNPs. By experiments and numerical simulations, important parameters that can affect the excitation pulse duration response of UCNPs were identified and their effects were analyzed. Our study shows that a systematic variation of the excitation pulse duration can be very informative and have important implications in the characterization and applications of UCNPs, such as in the assignment of transitions of different emission peaks, for bioimaging, and in luminescent nanothermometry.

The response of UCNPs to pulsed excitation can be investigated and examined in many ways, varying several different parameters of the pulsed excitation (e.g., pulse duration, duty cycle, and frequency) and monitoring and/or quantifying different changes in the upconversion luminescence (UCL) signal. In this work, we, following the TRAST concept, applied on organic fluorophores, confined ourselves to study how the average UCL intensity, ⟨UCL(λ,*w*)⟩, for different emission bands of UCNPs at wavelength λ varied with pulse width *w* while keeping the duty cycle η of the square-wave pulse constant (see Figure 1a). During the measurements, UCNPs were irradiated by square-wave modulated 980 nm laser light, and the UCL intensity was recorded by a spectrometer with a sufficiently long exposure time to properly average the UCL photons emitted over multiple periods. The OFF time in a pulse period was carefully controlled to allow UCNPs to completely relax to the ground state after the photoexcitation during the ON time, to eliminate pile-up effects, i.e., remaining excited state populations between adjacent excitation pulses. The recorded UCL intensity was normalized to approach unity at long pulse widths by
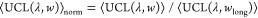
1and the dependence of ⟨UCL(λ,*w*)⟩_norm_ on *w* was plotted to yield an excitation pulse duration response curve, also termed a TRAST curve in previous studies of organic fluorophores.^11^

**Figure 1:**
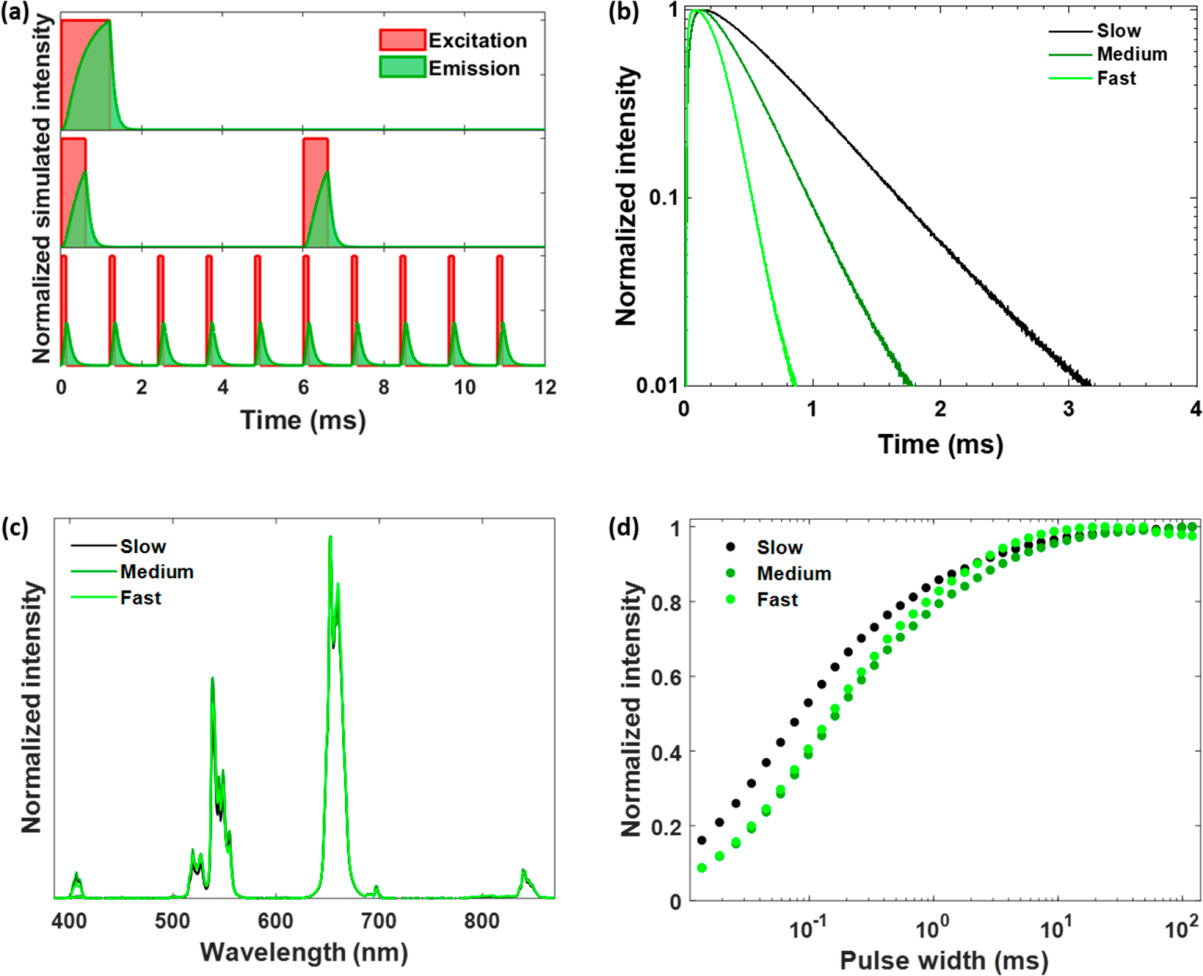
(a) Schematic diagram of the excitation pulse duration response of an upconversion system (red for excitation and green for upconversion luminescence). A 10% duty cycle is used for a better visual presentation. (b) Upconversion luminescence rise–decay profiles at 540 nm for the three Yb^3+^-Er^3+^-codoped upconversion nanoparticle samples with different kinetics (Figure S1) following a short-pulse excitation (10 μs) at 980 nm. (c) Upconversion luminescence spectra of the three Yb^3+^-Er^3+^-codoped upconversion nanoparticle samples with different kinetics (Figure S1) under continuous-wave 980 nm excitation. (d) Excitation pulse duration response of the 540 nm upconversion luminescence band for three Yb^3+^-Er^3+^-codoped upconversion nanoparticle samples with different kinetics (Figure S1), under excitation by a modulated 980 nm laser (0.2% duty cycle). Each data point is the average of three measurements. The standard deviation is much smaller than the symbol size. Measurements were performed at room temperature with an average excitation power density of 56 mW cm^–2^.

This approach was first applied to three Yb^3+^-Er^3+^-codoped NaYF_4_ nanoparticle samples, synthesized using previously reported protocols with minor modifications.^19,20^ These nanoparticles have different sizes and shapes, as characterized by transmission electron microscopy (TEM) (Figure S1), due to different synthesis conditions (see section 1 of the Supporting Information). Accordingly, these nanoparticles have different UCL kinetics under short-pulse 980 nm excitation, with the smallest UCNPs having faster kinetics and the larger core–shell UCNPs having slower kinetics (see Figure 1b and Figure S2). These three groups of nanoparticles are hereafter denoted as the “Fast”, “Medium”, and “Slow” groups, referring to their different UCL kinetics. They give typical UCL spectra of the Er^3+^ ions under continuous-wave 980 nm excitation (Figure 1c). Employing a simple lab-made setup (see details in section 2 of the Supporting Information), the excitation pulse duration responses of these UCNPs were measured by varying the excitation pulse width, while using a constant 0.2% duty cycle. The results for the Er^3+^ emission band at 540 nm are presented in Figure 1d. In the excitation pulse duration response curves, ⟨UCL(λ,*w*)⟩_norm_ generally exhibits a relatively fast increase in the short-pulse width range and then gradually reaches a plateau at longer pulse durations. Such a general trend can be explained by the fact that UCL originates from high-lying lanthanide excited states, which need time to be pumped, and thus, the upconversion emission system operates with a lower quantum efficiency at a non-steady state than at the steady state.^13,21^ It should be noted here that the selected 0.2% duty cycle was small enough to avoid pile-up effects (see the discussion in section S4 of the Supporting Information). It is noticeable in Figure 1d that the excitation pulse duration responses of the three different UCNP samples show significant differences and that for UCNPs with fast kinetics a slight decrease in ⟨UCL(λ,*w*)⟩_norm_ can be noticed for longer values of *w* (>10 ms).

The excitation pulse duration response curves for other UCL bands of Er^3+^ ions of the three UCNP samples were also observed, together with their impulse responses, as shown in Figures S2, S5, and S6. There were no significant pile-up effects found with a duty cycle of 0.2% (Figure S3). However, with a duty cycle of 2%, a significant pile-up was observed in all emission bands at shorter values of *w* (Figures S4 and S5).

To get a hold of which parameters can affect the UCL response to different excitation pulse durations, we performed numerical simulations based on a simplified two-photon upconversion model (Figure S7), representing a Yb^3+^-sensitized two-photon UCL emission. The model is described in detail in section S5 of the Supporting Information. The effects of the lifetime of the sensitizer excited state, the lifetime of the intermediate state of the activator ion, the lifetime of the UCL emitting state of the activator ion, the energy transfer rates for the two energy transfer steps involved in the upconversion process, and the rate for a possible cross-relaxation process were investigated by respective simulations, while keeping other parameters constant. Small values for the duty cycle (Table S2) were selected to fully avoid the interference of the pile-up effect (Figure S8a). The calculated results show that the response in the modeled two-photon UCL, ⟨UCL(λ,*w*)⟩_norm_, is more sensitive to changes in the lifetime of the intermediate state of the activator ion and the energy transfer rates, and less to other parameters (Figure S8c–h). In particular, this response is very insensitive to the change in the lifetime of the UCL-emitting state (Figure S8e). This agrees with the results depicted in Figure 1d, where, given that the apparent decay times of the 540 nm band under short-pulse 980 nm excitation for the UCNPs with fast and medium kinetics are very different, they do not lead to very different excitation pulse duration response curves.

To gain more insight into the excitation pulse duration response of UCL to understand especially its insensitivity or sensitivity to different parameters, we performed in-depth theoretical analysis based on the rate equation model for a standard two-photon upconversion process (see details in sections S5 and S6 of the Supporting Information). By adapting a weak-excitation approximation to further simplify the question, we can describe the excitation pulse duration response by (sections S5 and S6 of the Supporting Information):
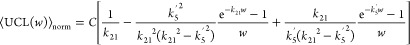
2where *C* is a constant, *k*_21_ is the decay rate of the intermediate state of the activator (Er^3+ 4^I_11/2_), and *k*_5_^′^ is the effective decay rate of the excited state of the sensitizer (Yb^3+ 2^F_5/2_), including the effect of the radiative decay rate (*k*_54_) and energy transfer rate (*k*_52_*n*_1_) (referring to eq 5 of the Supporting Information). Equation 2 indicates that the shape of ⟨UCL(*w*)⟩_norm_ does not show dependence on the decay rate (or its inverse lifetime) of the UCL emitting state, agreeing well with the simulation result shown in Figure S8e. Equation 2 also predicts that ⟨UCL(*w*)⟩_norm_ is largely determined by *k*_21_, the decay rate of the intermediate state of the activator (Er^3+ 4^I_11/2_), completely in line with the simulation result shown in Figure S8d. In addition, the dependence of ⟨UCL(*w*)⟩_norm_ on *k*_5_^′^, the effective decay rate of the excited state of the sensitizer (Yb^3+ 2^F_5/2_), can well explain the effects of the radiative decay rate (or its inverse lifetime) of the Yb^3+ 2^F_5/2_ state and the rate of transfer of energy from this state to the Er^3+^ ground state (^4^I_15/2_), while the effect of the latter is even larger than that of the former (Figure S8c,f), which can be explained by the difference in their magnitudes. Noting that typically *k*_5_^′^ ≫ *k*_21_, according to eq 2, the shape of ⟨UCL(*w*)⟩_norm_ may be dominantly affected by rate constant *k*_21_. It should also be noted that under the used weak-excitation approximation, i.e., neglecting the contribution of the second-step energy transfer process [from the Yb^3+ 2^F_5/2_ state to the Er^3+ 4^I_11/2_ state (ETU2 in Figure S7)] to the deactivation of the Yb^3+ 2^F_5/2_ and Er^3+ 4^I_11/2_ states, the effect of the rate for this process on ⟨UCL(*w*)⟩_norm_ is not reflected in eq 2. Taken together, the excitation pulse duration response of UCL provides a facile approach to sense the information about the key intermediate states involved in the upconversion process and their population kinetics.

We point out that the limitation of our simulation studies and theoretical analysis, based on a model of the upconversion process, is obvious. In a real upconversion system, much more complicated processes could happen as disclosed in previous works, e.g., complex energy transfer and cross-relaxation pathways,^22−24^ surface coupling,^23,25,26^ and the thermal effect.^27^ These processes would more or less have an effect on the excitation pulse duration response of the UCL and accordingly change the information that we can get from this response. For instance, in the simulations, we could not reproduce the decrease in ⟨UCL(λ,*w*)⟩_norm_ for longer values of *w* (>10 ms), as experimentally found in the case of the 540 nm UCL band of the “Fast” group of UCNPs (Figure 1d). With regard to this specific behavior, we speculate that it was due to the ignition of the higher-order energy transfer upconversion process reinforced by a long excitation pulse duration, similar to that reported in a previous work.^18^

Here, it is instructive to further discuss the possibility of extracting the important parameter values characterizing the UCL from the experimental excitation pulse duration response data. For comparison, in TRAST spectroscopy studies, the same modulation–detection approach has been successfully applied to quantify dark state transitions in organic fluorophores for bioimaging and biosensing purposes.^11,29,30^ While our experimental data show that this approach is also feasible for UCNPs, the larger number of states involved and the nonlinear response of UCNPs significantly increase the model complexity and add difficulty in retrieving the rate parameter values. On the contrary, a consequence of this added complexity is also that the excitation pulse duration response of UCNPs can be used as an optical encoding/decoding technique for encryption applications.^32,33^ It allows the possibility of information being encrypted within different groups of UCNPs showing the same emission color (upon NIR excitation) but having different excitation pulse duration responses. Distinguishing different UCNPs by their responses to excitation pulse durations thus opens an almost infinite internal encoding space. In addition, because our analysis requires that only the time-averaged UCL intensity (⟨UCL(λ,*w*)⟩_norm_) needs to be detected, multipixel detectors with low time resolution can be used for decoding. This strongly reduces the instrumental requirements, increases applicability, and makes possible a faster reading than when point-like detectors must be used.

It is known that the luminescence kinetics of UCNPs exhibit a significant dependence on the excitation intensity.^34−36^ A high excitation intensity favors the populations of high-lying energy levels and can lead to significantly different intensity ratios between different emission bands compared to under low-intensity excitation. We have studied the effect of excitation intensity on the excitation pulse duration response of UCL. Figure 2a shows how ⟨UCL(λ,*w*)⟩_norm_ at λ = 540 nm for the Yb^3+^-Er^3+^-codoped UCNPs with slow kinetics varies with *w*, and how the average excitation intensity, *I*_exc_, influences the excitation pulse duration response curves significantly, where a higher *I*_exc_ generally increased the UCL intensity at all values of *w* and reduced the variation ranges of the response curves. This trend could also be reproduced by numerical simulations (Figure S8b). Further analyzing the *I*_exc_ dependence in the excitation pulse duration response curves recorded from the 540 nm UCL band (Figure 2b) revealed that the slope of the UCL versus *I*_exc_ increases with a decrease in pulse width, which is in large contrast to the corresponding dependence under continuous-wave excitation (Figure 2c). Similar effects caused by variation of *I*_exc_ were also found for other UCL bands (Figure S9). This implies that the use of short excitation pulses better preserves the nonlinear optical responses of UCNPs and may be particularly useful for optical sectioning and super-resolution microscopy techniques, which specifically benefit from the nonlinearity of the UCNPs.^37−40^

**Figure 2:**
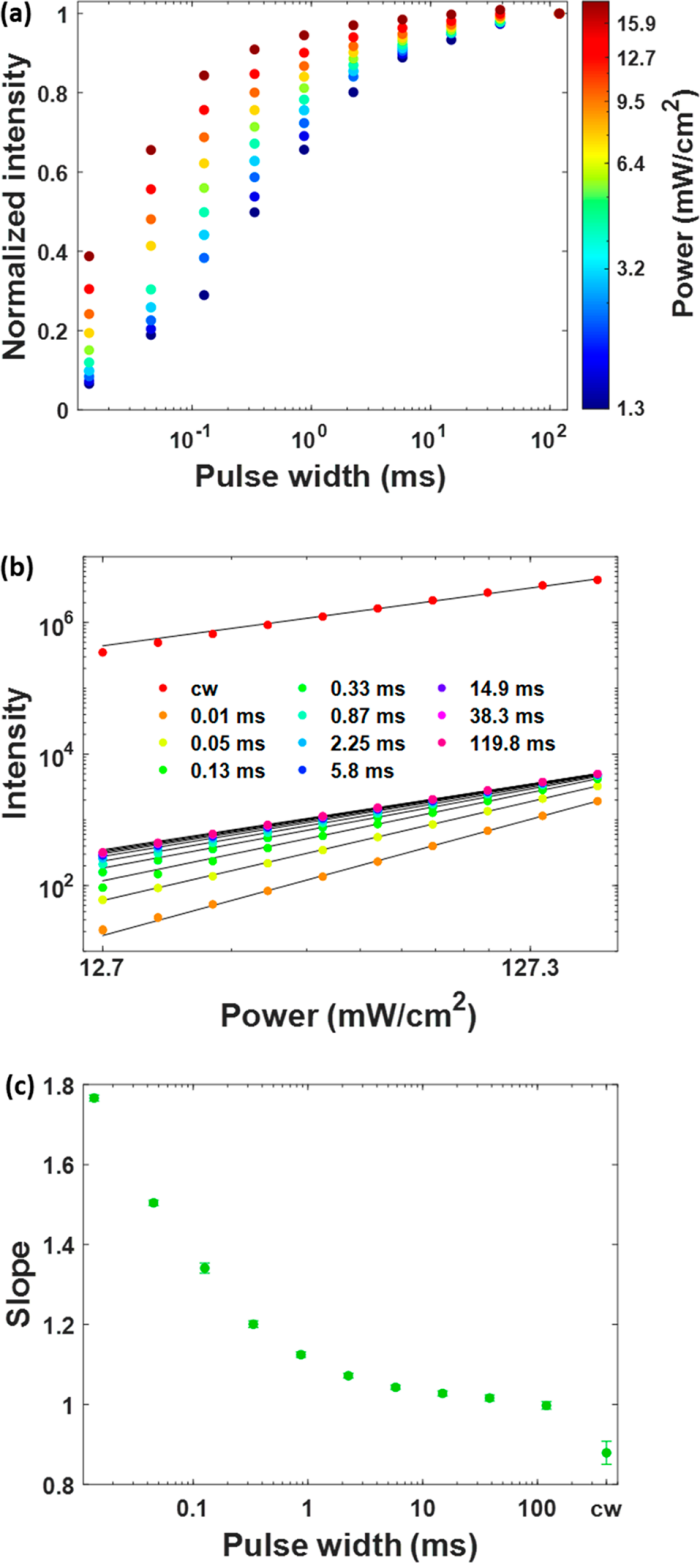
(a) Normalized emission intensity, ⟨UCL(λ,*w*)⟩_norm_, at λ = 540 nm for the Yb^3+^-Er^3+^-codoped upconversion nanoparticles with slow kinetics, plotted vs *w* (0.2% duty cycle), and recorded under different *I*_exc_ values by a 980 nm modulated laser. Each data point is the average of three measurements. The standard deviation is much smaller than the point size. (b) Intensity of the green emission band of Er^3+^ at 540 nm for the Yb^3+^-Er^3+^-codoped upconversion nanoparticles with slow kinetics as a function of *I*_exc_, under 980 nm pulsed excitation with different values of *w* (0.2% duty cycle) or continuous-wave excitation. Black lines are the fits to ⟨UCL(λ,*w*)⟩ ∝ *I*_exc_^*n*^. Each data point is the average of three measurements. The standard deviation is much smaller than the symbol size. (c) Variation of the slope (parameter *n*) from the fitting of ⟨UCL(λ,*w*)⟩ vs *I*_exc_ in panel (b). The average value of *R*^2^ is 0.9995 ± 0.0012. Error bars obtained from a 95% confidence interval of *n* from fitting to ⟨UCL(λ,*w*)⟩ ∝ *I*_exc_^*n*^.

We further examined the response in ⟨UCL(λ,*w*)⟩ to excitation pulse duration *w* over the full UCL spectrum (400–900 nm) of the Yb^3+^-Er^3+^-codoped UCNPs with slow kinetics. The UCL spectrum of this UCNP sample shows a noticeable difference under pulsed excitation with different pulse widths (Figure 3b). The different UCL emission bands can be assigned to transitions of Er^3+^ ions shown in Figure 3a. It is known that different emission bands from the same UCNPs may have distinct or the same kinetics, depending on if they refer to a transition originating from a different or the same higher energy level(s), respectively. Because the excitation pulse duration response is closely related to UCL kinetics, we propose that it can be used to identify the emission peaks originating from the same higher-energy state. For instance, the emission peaks at 540 and 840 nm both originate from the same ^4^S_3/2_ energy state (Figure 3a), and thus, they exhibit almost identical excitation pulse duration responses (Figure 3c). In addition, the 410 and 555 nm emission peaks, originating from transitions from the same ^2^H_9/2_ energy level, also show very similar excitation pulse duration responses (Figure 3c). Interestingly, the 520 and 540 nm emissions, well known to originate from transitions from two strongly thermally coupled states (^2^H_11/2_ and ^4^S_3/2_),^41^ show noticeable differences in their excitation pulse duration response curves. These differences could be a result of the differences in the coupling channels of these two states with other Yb^3+^ or Er^3+^ states. For example, it was reported that the Er^3+ 4^S_3/2_ state could be involved in a cross-relaxation process between two Er^3+^ ions (^4^S_3/2_ + ^4^I_15/2_ → ^4^I_13/2_ + ^4^I_11/2_),^42^ an energy transfer process involving Er^3+^ and Yb^3+^ [^4^S_3/2_ (Er^3+^) + ^2^F_7/2_ (Yb^3+^) → ^4^I_13/2_ (Er^3+^) + ^2^F_5/2_ (Yb^3+^)],^43^ and an excited state absorption process of Er^3+^ (^4^S_3/2_ → ^2^G_7_),^42^ but not the Er^3+ 2^H_11/2_ state. This requires further investigation.

**Figure 3:**
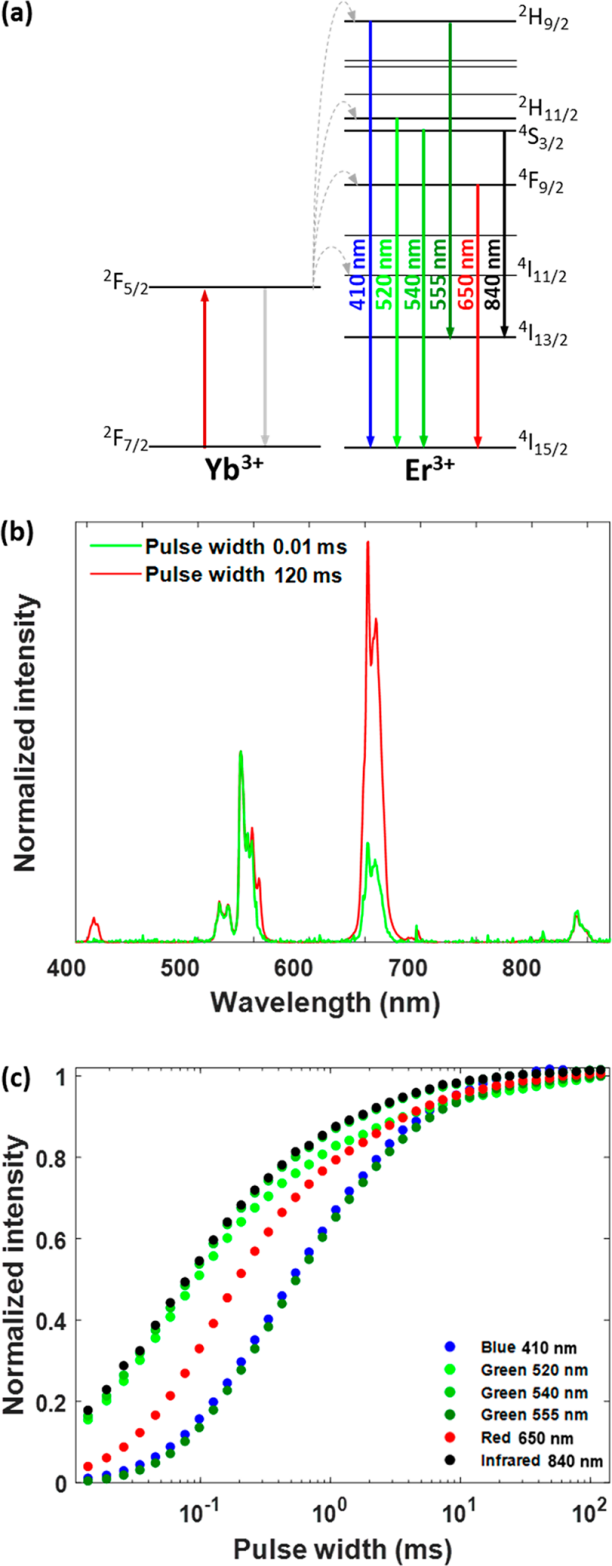
(a) Energy level diagram of the Yb^3+^-Er^3+^ upconversion system showing the transitions of upconversion luminescence peaks of interest. (b) Normalized (to the intensity at the 540 nm peak) emission spectra of Yb^3+^-Er^3+^-codoped upconversion nanoparticles with slow kinetics under 980 nm pulsed excitation with pulse widths of 0.01 ms (green) and 120 ms (red) (0.2% duty cycle). (c) Dependence of the intensity of different emission peaks of Yb^3+^-Er^3+^-codoped upconversion nanoparticles with slow kinetics on the excitation pulse width of a 980 nm square-wave excitation (0.2% duty cycle). Each data point is the average of three measurements. The standard deviation is much smaller than the symbol size. Measured at room temperature with an average excitation power density of 56 mW cm^–2^.

We also performed some excitation pulse duration response studies of NaYF_4_:Yb^3+^,Tm^3+^ UCNPs, with multiple well-assigned UCL bands, some of which are known to originate from transitions from the same higher state (Figure S13). An appropriate duty cycle was used for each emission band to avoid pile-up effects (Figure S13i). Similar to the case of Er^3+^-based UCNPs, the emission peaks from transitions from the same higher state, e.g., the 475 and 645 nm peaks from the ^1^G_4_ energy level and the 360, 450, and 740 nm peaks from the ^1^D_2_ energy level, exhibit very similar excitation pulse duration response curves (Figure S13j).

This observation made us realize that the distinct excitation pulse duration responses of spectrally overlapping emission peaks are actually coming from different transitions and indicate the possibility of suppressing a certain emission peak by using a proper square-wave excitation pulse width. This may have important implications for biosensing applications of UCNPs. One example shown here is given by the two green emission peaks of Er^3+^ at 540 and 555 nm, originating from the ^4^S_3/2_ → ^4^I_15/2_ and ^2^H_9/2_ → ^4^I_13/2_ transitions, respectively (Figure 3a). As indicated by their distinct excitation pulse duration response curves (Figure 3c), it is predictable that the emission peak at 555 nm can be suppressed while still exciting the main emission band at 540 nm, if the UCNPs are excited by square-wave excitation with proper parameters (see Figures 3b and 4a,b). Because the 555 nm emission peak comes from a transition from a higher-lying energy level, its intensity usually increases faster with an increase in *I*_exc_. However, we found that the effectiveness of using the short-pulse excitation approach to suppress the intensity of the 555 nm emission peak relative to the 540 nm main band remains in a broad range of average excitation densities, 12–183 mW cm^–2^ (the limits of our instrument), as shown in Figure S12a. Nevertheless, the suppression of the contribution of the 555 nm emission peak to the main green emission band may have a slightly reduced effectiveness if the duty cycle of the modulated excitation is not sufficiently low to avoid pile-up effects (Figure S12c,d).

**Figure 4:**
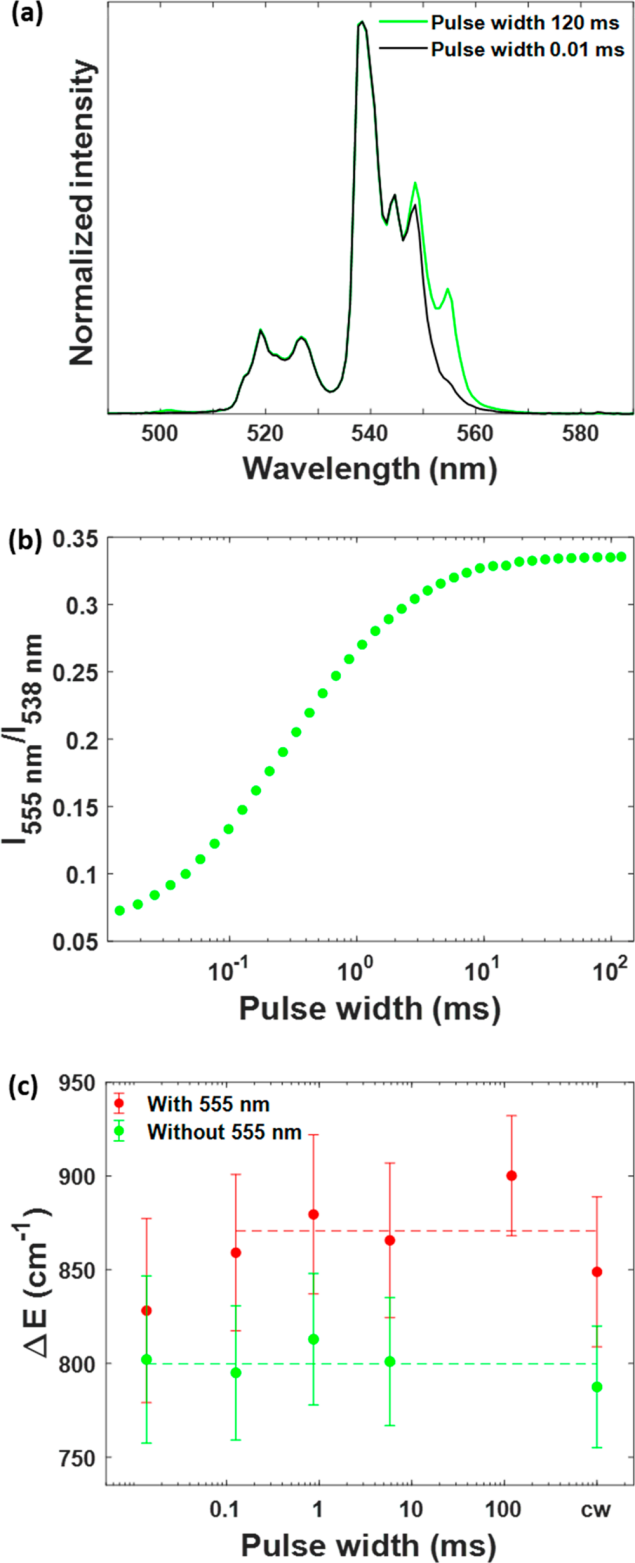
(a) Normalized green emission bands of Yb^3+^-Er^3+^-codoped upconversion nanoparticles with slow kinetics under 980 nm pulsed excitation with pulse widths of 0.01 ms (black) and 120 ms (green) (0.2% duty cycle). Measured at room temperature with an average excitation power density of 56 mW cm^–2^. (b) Dependence of the intensity ratio of the emission peaks at 555 and 538 nm of Yb^3+^-Er^3+^-codoped upconversion nanoparticles with slow kinetics on the excitation pulse width of the 980 nm modulated laser (0.2% duty cycle). Each data point is the average of three measurements. The standard deviation is much smaller than the symbol size. Measured at room temperature with an average excitation power density of 56 mW cm^–2^. (c) Fitted energy mismatch (*ΔE*) between the ^2^H_11/2_ and ^4^S_3/2_ energy levels of Er^3+^ ions using intensity ratio data measured under 980 nm continuous-wave and pulsed excitations, including (red) or excluding (green) the 555 nm emission peak in the integrated area. Error bars were obtained from the 95% confidence interval of *ΔE* in the fitting to the Boltzmann distribution *I*_520_/*I*_540_ ∝ exp(−Δ*E*/*k*_B_*T*). The average value of *R*^2^ is 0.987 ± 0.004. See more details in section S8 of the Supporting Information.

The possibility of suppressing the emission peak at 555 nm of Er^3+^ ions is of high interest for nanothermometry applications using Yb^3+^-Er^3+^-codoped UCNPs, where the intensity ratio between the sub-bands at 520 (^2^H_11/2_ → ^4^I_15/2_) and 540 nm (^4^S_3/2_ → ^4^I_15/2_) is frequently used as the temperature indicator. The principle is that the populations at the ^2^H_11/2_ and ^4^S_3/2_ states follow a Boltzmann distribution because they are thermally coupled and thus that this intensity ratio is related to the temperature by the relationship *I*_520_/*I*_540_ ∝ exp(−Δ*E*/*k*_B_*T*), where *ΔE* is the energy mismatch of the two states and *k*_B_ is the Boltzmann constant.^44,45^ In practical nanothermometry applications, usually low-resolution spectral discriminating devices (such as band-pass filters) are used due to cost considerations, making it difficult to block the 555 nm emission peak from the 540 nm band. As a result, its presence will cause non-negligible errors in the temperature evaluation. Several recent works have been devoted to studying the conditions under which this emission peak appears and to find strategies to prevent it.^46−50^Figure 4c shows the values of *ΔE* obtained by fitting the experimental *I*_520_/*I*_540_ intensity ratio measured under continuous-wave and short-pulse excitation as a function of temperature (Figure S11). If the contribution of the 555 nm emission peak is not considered, all of the data sets returned similar values for *ΔE*, and the average of the obtained values should then be a good approximation of the expected value. However, if the interference of the 555 nm emission peak is present, *ΔE* is overestimated when using the data measured under continuous-wave and long excitation pulse widths. The fitted *ΔE* value using the data under short-pulse excitation is closer to the expected value. This suggests that the use of short-pulse excitation improves the reliability of this thermometry readout, based on the intensity ratio of the green UCL sub-bands of Yb^3+^-Er^3+^-codoped UCNPs, and this strategy may also be useful for other UCNP sensors with spectral overlap issues.^51^

Lanthanide-doped upconversion nanoparticles (UCNPs), operating as optical kinetic systems, exhibit fascinating features in response to temporal excitation modulations. As particularly studied in this work, the response of the upconversion luminescence of UCNPs to the pulse width in square-wave excitation shows a complex dependence on the fundamental photophysical parameters characterizing the upconversion mechanisms and on the excitation intensity. As implicated by the results presented in this work, the square-wave excitation modulation technique offers many opportunities for investigating and applying UCNPs, e.g., for anticounterfeiting, identifying emission peaks originating from transitions from the same excited state, super-resolution microscopy, and reliable luminescence nanothermometry.

## Supporting Information Available

Additional experimental details, materials and methods, and supporting data on characterization of nanomaterials, effect of the duty cycle, rate equation simulations, theoretical analysis, effect of excitation intensity, effect of temperature, Er^3+^ emission band ratios, and application to Yb^3+^-Tm^3+^-codoped nanoparticles (PDF)
