# Supplementary material for: Excitation Pulse Duration Response of Upconversion Nanoparticles and Its Applications
Source: J Phys Chem Lett. 2022 Nov 29;13(48):11208–15. doi: 10.1021/acs.jpclett.2c03037 (PMC9743204; doi:10.1021/acs.jpclett.2c03037)
Supplement: Supplementary file 1 — jz2c03037_si_001.pdf [file jz2c03037_si_001.pdf]

Supporting Information

Excitation Pulse Duration Response of Upconversion Nanoparticles and Its Applications

Lucía Labrador-Páez,[a] Uliana Kostiv,[a] Qingyun Liu,[b] Yuanyuan Li,[c] Hans Ågren,[d] Jerker Widengren,[a] and Haichun Liu[a]*

[a] Department of Applied Physics, KTH Royal Institute of Technology, SE-10691 Stockholm, Sweden.

[b] Department of Theoretical Chemistry and Biology, KTH Royal Institute of Technology, SE-10691 Stockholm, Sweden.

[c] Wallenberg Wood Science Center, Department of Fibre and Polymer Technology, KTH Royal Institute of Technology, Stockholm, Sweden.

d Department of Physics and Astronomy, Uppsala University, Uppsala, SE-75120 Sweden

*Corresponding author. E-mail: haichun@kth.se

S1. Synthesis and characterization of nanomaterials

S2. Spectroscopical characterization setup

S3. Spectroscopical characterization

S4. Effect of duty cycle

S5. Rate equations simulations

S6. Theoretical analysis of the excitation pulse duration response of two-photon upconversion luminescence

S7. Effect of excitation intensity

S8. Effect of temperature

S9. Er^{3+} emission bands ratios

S10. Application to Yb^{3+}-Tm^{3+}-codoped nanoparticles

S1. Synthesis and morphological characterization of nanomaterials

Chemicals and Materials

Anhydrous yttrium(III), ytterbium(III), erbium(III), and thulium(III) chlorides (99%), oleic acid (90%), octadec-1-ene (90%), methanol, sodium hydroxide, ammonium fluoride were purchased from Sigma-Aldrich (St. Louis, MO, USA). Cyclohexane, ethanol, and microscope slides were purchased from VWR (Radnor, PA, USA).

Synthesis of core $NaYF_4:Yb^{3+},Er^{3+}$ and core $NaYF_4:Yb^{3+},Tm^{3+}$ nanoparticles

The core nanoparticles were synthesized according to previously published reports.[1, 2] Into a 100-mL three-neck round-bottom flask, 1 mmol of lanthanide chlorides, oleic acid (6 mL), and octadec-1-ene (15 mL) were mixed. To prepare $NaYF_4:Yb^{3+},Er^{3+}$, 0.78 mmol YCl_3, 0.2 mmol $YbCl_3$, and 0.02 mmol $ErCl_3$ were used. To prepare $NaYF_4:Yb^{3+},Tm^{3+}$, 0.795 mmol YCl_3, 0.2 mmol $YbCl_3$ and 0.005 mmol $TmCl_3$ were used. In each case, the mixture was heated at 160°C for 30 min with stirring under continuous Ar flow until the mixture became homogeneous, and then it was cooled down to room temperature. After cooling, a methanolic solution of NaOH (2.5 mmol) and NH_4F (4 mmol) was added to the reaction mixture. The reaction temperature gradually increased from room temperature to 300°C. Different reaction time was used to prepare different nanoparticles (Table S1). Next, the mixture was naturally cooled down to room temperature, and the $NaYF_4:Yb^{3+},Er^{3+}$ or $NaYF_4:Yb^{3+},Tm^{3+}$ nanoparticles were collected by centrifugation (3.461 g, 15 min). After centrifugation, the solution above a white pellet (precipitate of nanoparticles) was discarded and the nanoparticles were dispersed in cyclohexane (10 mL), precipitated by ethanol (5 mL), and centrifugated (3.461 g, 15 min). This step was repeated one more time and, finally, the nanoparticles were dispersed in cyclohexane.

Synthesis of core-shell $NaYF_4:Yb^{3+}/Er^{3+}@NaYF_4$ nanoparticles

The core-shell $NaYF_4:Yb^{3+},Er^{3+}@NaYF_4$ nanoparticles were synthesized according to earlier published reports.[1, 2] 0.05 mmol of yttrium chloride, oleic acid (6 mL), and octadec-1-ene (15 mL) were mixed and heated at 160°C for 30 min under Ar flow to get a homogeneous yellowish solution and cooled down. After cooling, the core $NaYF_4:Yb^{3+},Er^{3+}$ nanoparticles, a methanolic solution of NaOH (1.25 mmol) and NH_4F (2 mmol) were added to the reaction mixture and heated gradually to 300°C for 1 h. The mixture was cooled down to room temperature and the core-shell nanoparticles were collected by centrifugation (3,461 g, 15 min). After centrifugation, the top solution was discarded and the core-shell nanoparticles left were dispersed in cyclohexane (10 mL), precipitated by ethanol (5 mL), and centrifugated (3,461 g, 15 min). This step was repeated one more time and, finally, the core-shell nanoparticles were dispersed in cyclohexane.

Table S1. Different reaction times to prepare core and core-shell nanoparticles of different shapes. The reaction temperate was kept constant at 300°C for all nanoparticle synthesis.

Sample Name	Material	Core synthesis time	Shell synthesis time
	$NaYF_4:Yb^{3+},Tm^{3+}$	1.5 h	-
Fast	$NaYF_4:Yb^{3+},Er^{3+}$	0.5 h	-
Medium	$NaYF_4:Yb^{3+},Er^{3+}$	1.0 h	-
Slow	$NaYF_4:Yb^{3+},Er^{3+}@NaYF_4$	1.5 h	1 h

Morphological characterization of Yb^{3+}-Er^{3+}-codoped nanoparticles

The nanoparticles were imaged by transmission electron microscopy (Talos 120C G2, Thermo Fisher Scientific, operating at 120 kV). From the analysis of those images, their size distribution

could be obtained (see Figure S1). Their average size is 55 ± 4 nm diameter and 53 ± 6 nm thickness for the hexagonal disks named as slow nanoparticles, 33.8 ± 1.5 nm width and 52.1 ± 1.5 nm length for the elongated rods named as medium nanoparticles, and 32.6 ± 2.0 nm width and 37.4 ± 1.0 nm length for the less elongated rods named as fast nanoparticles (names are due to their spectroscopic kinetics, see Figure S2).

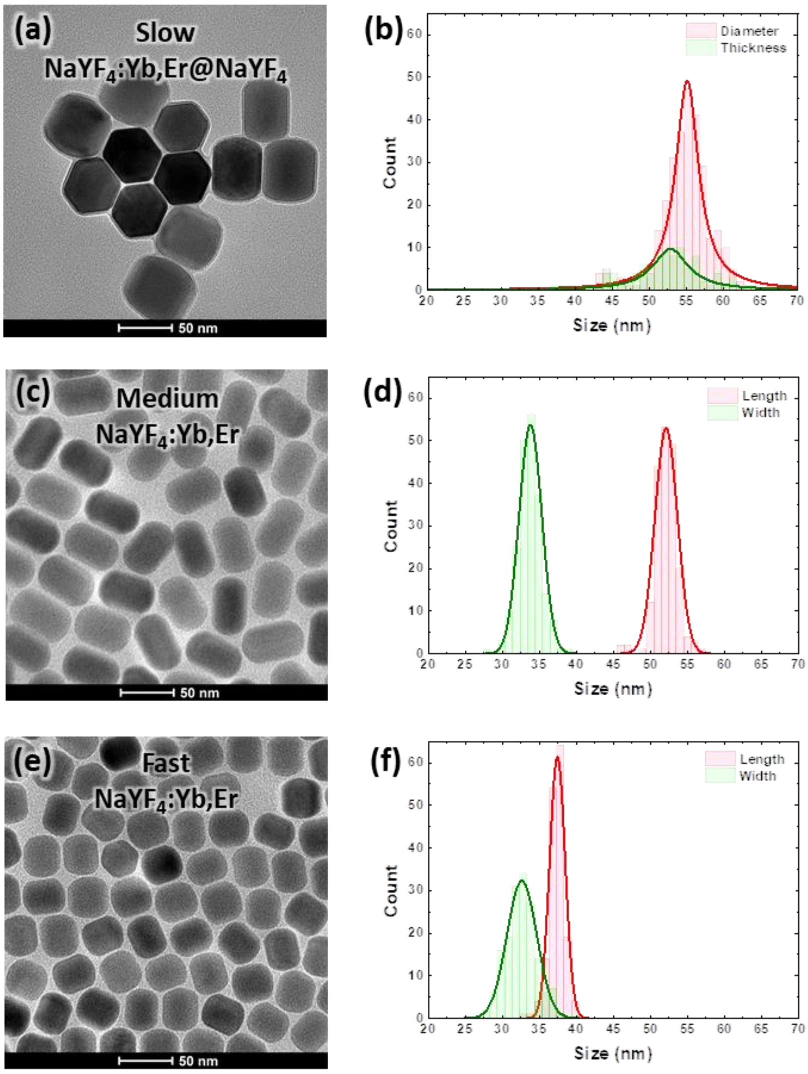

Figure S1. Transmission electron microscopy (TEM) images (scale bars 50 nm) and size histograms (obtained from the analysis of several TEM images) of **(a)-(b)** $NaYF_4$:Yb,Er@$NaYF_4$ nanoparticles with hexagonal disk shape (with slow kinetics), **(c)-(d)** $NaYF_4$:Yb,Er nanoparticles with more elongated rod shape (with medium kinetics), and **(e)-(f)** $NaYF_4$:Yb,Er nanoparticles with less elongated rod shape (with fast kinetics), respectively.

S2. Spectroscopic characterization setup

The kinetics of the upconversion nanoparticles (UCNPs) were explored by their response to square-wave modulated excitation at 975 nm. For that purpose, a 975 nm laser diode (L9418-42, Hamamatsu) was modulated by an arbitrary waveform generator (BK4053B, B&K Precision) and focussed by a lens (4 cm focal lens, Thorlabs). The emission of the UCNPs was then collected by a lens (6 cm focal lens, Thorlabs), filtered (FESH0950 or FESH0700, Thorlabs), and collimated

into an optical fiber by a lens (6 cm focal lens, Thorlabs). The optical fiber transmitted the filtered luminescence to a spectrometer (QEPro, Ocean Optics). The average laser power was controlled by the function generator and measured by a power meter (PM100D and S121C, Thorlabs). The temperature was modified by a heating plate (IKA RCT basic) and controlled by a reflectivity-based thermometer.

S3. Spectroscopic characterization

The three types of UCNPs were deposited and dried on a microscope slide. Then they were spectroscopically characterized, obtaining their upconversion emission spectra under excitation at 975 nm (Figure 1b). Moreover, the kinetics of their different emission bands were preliminary characterized by obtaining the intensity decay under pulsed excitation at 975 nm (Edinburgh Instruments FS5). The results, included in Figure S2, show that the three types of Yb^{3+}-Er^{3+}-codoped UCNPs have very different kinetics.

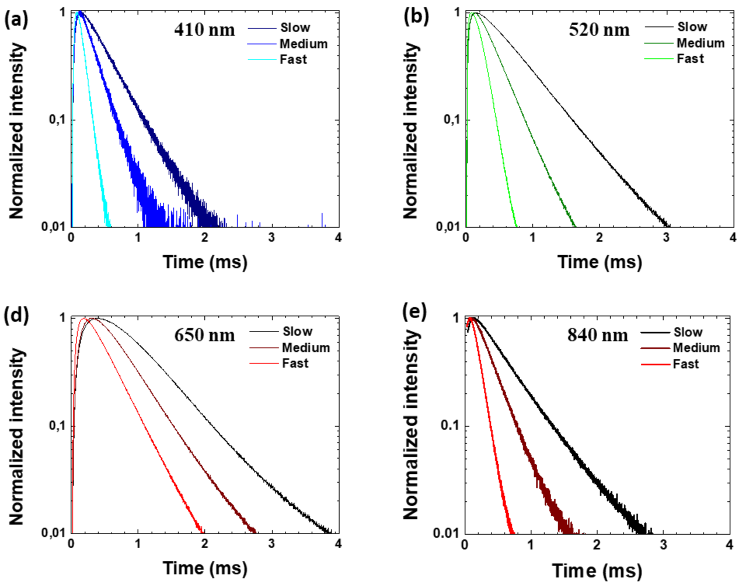

Figure S2. Intensity decay at **(a)** 410 nm ($^2H_{9/2} \rightarrow {}^4I_{15/2}$), **(b)** 520 nm ($^2H_{11/2} \rightarrow {}^4I_{15/2}$), **(c)** 540 nm ($^4S_{3/2} \rightarrow {}^4I_{15/2}$), **(d)** 650 nm ($^4F_{9/2} \rightarrow {}^4I_{15/2}$), and **(e)** 840 nm ($^4S_{3/2} \rightarrow {}^4I_{13/2}$) under pulsed excitation at 980 nm (pulse width 10 μs) for the Yb^{3+}-Er^{3+}-codoped UCNPs with different kinetics introduced in Figure S1.

S4. Effect of duty cycle

If the duty cycle (ratio of pulse width and period) of the square-wave modulation is not sufficiently low, the upconversion emission intensity emitted during one period may not have time to finish its decay before the start of the next period. Then, a pile-up effect would take place, artificially increasing the average emission intensity recorded. This effect is paramount at short excitation pulse widths, where a high duty cycle may alter the obtained value for the emitted intensity significantly. For that reason, a duty cycle test must be performed beforehand. Figure S3 shows the duty-cycle test for the Yb^{3+}-Er^{3+}-codoped UCNPs with different kinetics introduced in Figure S2, where the intensity emitted per excitation pulse is assessed for the shortest possible excitation pulse width and varying duty-cycle values. The normalized emission intensity per period increases when the duty cycle is too large (i.e., the period is too short in comparison with the excitation pulse width). From these tests, the duty cycle was set at 0.2% for Yb^{3+}-Er^{3+}-codoped

UCNPs. To illustrate this effect, the dependence of the different emission bands of Yb^{3+}-Er^{3+}-codoped UCNPs on the excitation pulse width was obtained at 0.2% and 2.0% duty cycles (Figure S4, Figure S5, and Figure S6). The larger duty cycle value generates an alteration of the results due to the pile-up effect, especially for the shortest excitation pulse widths and for the UCNPs with the slowest kinetics. The calculations plotted in Figure S8a also show the magnitude of the pile-up effect for a typical upconversion system.

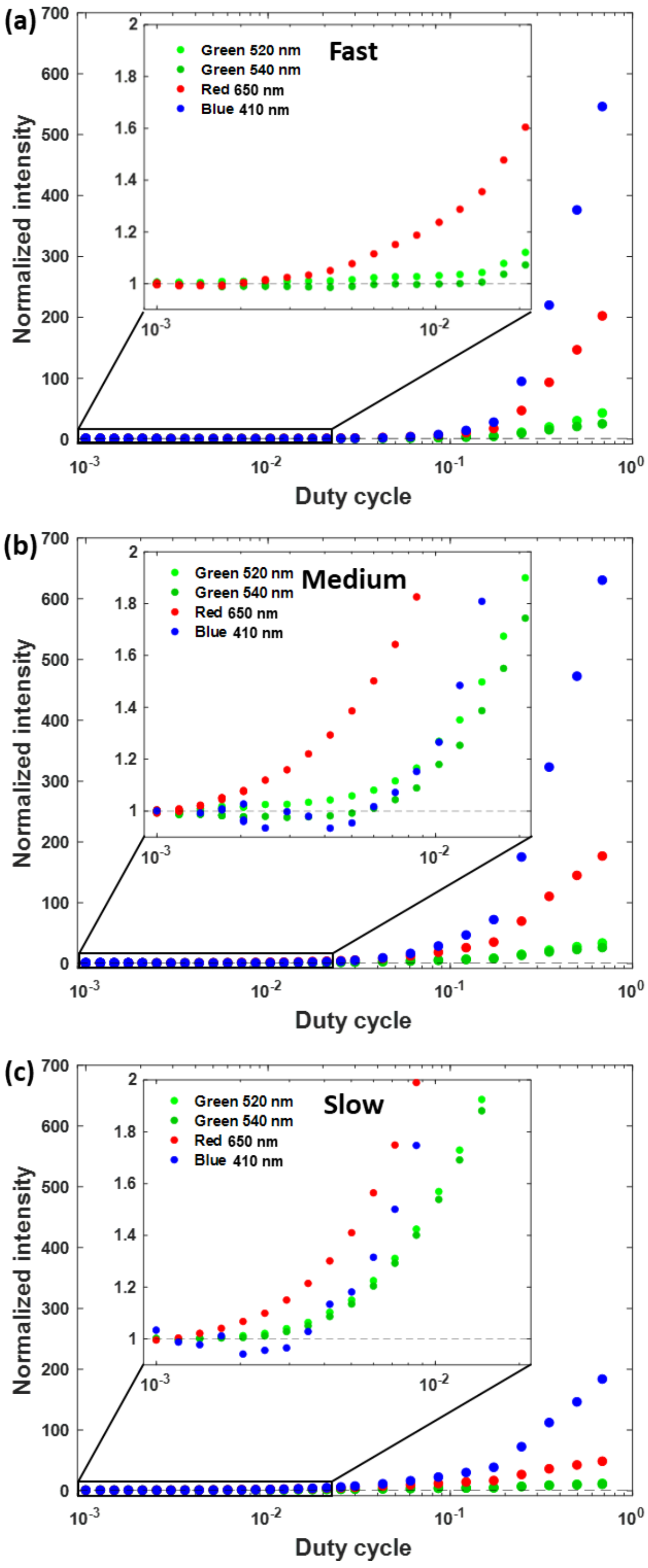

Figure S3. (a), **(b)**, and **(c)** Dependence of the emission intensities of different emission bands of Yb^{3+}-Er^{3+}-codoped UCNPs with different kinetics (see Figure S2) under modulated excitation at 980 nm with a fixed pulse width (0.013 ms) on the duty cycle of the modulation. Each data point is the average of at least three measurements. Standard deviation is much smaller than the symbol size. Insets are zooms of the region with lower duty-cycle values.

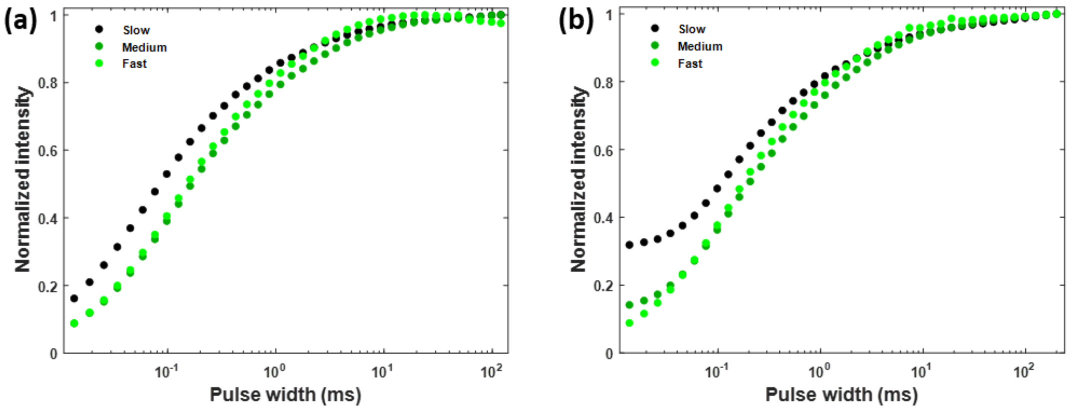

Figure S4. Dependence of the intensity of the green (540 nm) emission band of Yb^{3+}-Er^{3+}-codoped UCNPs with different kinetics (see Figure S2) on the excitation pulse width of the 980 nm modulated laser for duty cycle values of **(a)** 0.2% and **(b)** 2%, respectively. Each data point is the average of three measurements. Standard deviation is much smaller than the symbol size.

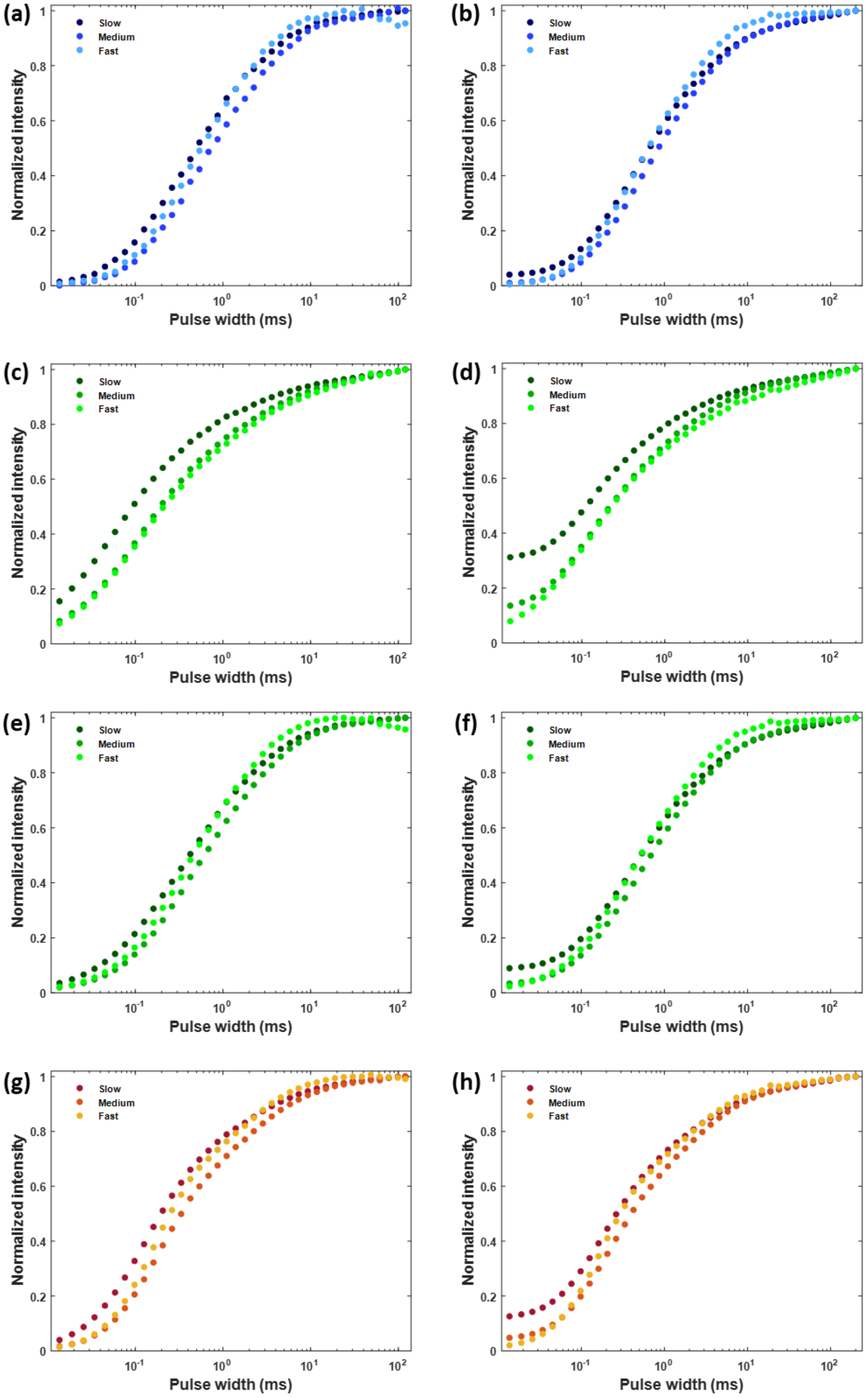

(a)
(b)
(c)
(d)
(e)
(f)
(g)
(h)
Slow
Medium
Fast
Normalized intensity
Pulse width (ms)
0
0.2
0.4
0.6
0.8
1
10^{-1}
10^{0}
10^{1}
10^{2}

Figure S5. Dependence of the intensities of blue, green at 520 nm, green at 555 nm, and red emission bands of Yb^{3+}-Er^{3+}-codoped UCNPs with different kinetics (see Figure S2) on the excitation pulse width of the 980 nm modulated laser for duty cycle values of **(a), (c), (e), (g)** 0.2% and **(b), (d), (f), (h)** 2%, respectively. Each data point is the average of three measurements. Standard deviation is much smaller than the symbol size.

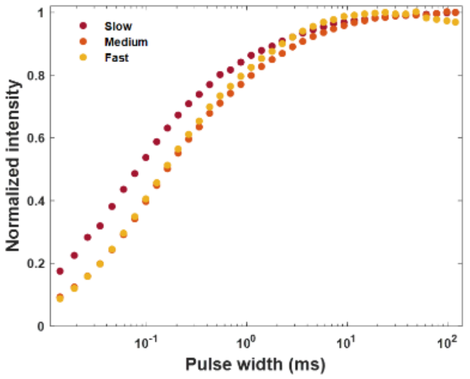

Figure S6. Dependence of the 840 nm emission band ($^4S_{3/2} \rightarrow {}^4I_{13/2}$) intensity on the excitation pulse width for Yb^{3+}-Er^{3+}-codoped UCNPs with different kinetics (see Figure S2) on the excitation pulse width of the 980 nm modulated laser (duty cycle 0.2%). Each data point is the average of three measurements. Standard deviation is much smaller than the symbol size.

S5. Rate equation simulations

Simulations of the dependence of the emitted intensity on the excitation modulation were performed using a simplified two-photon upconversion model depicted in Figure S7.

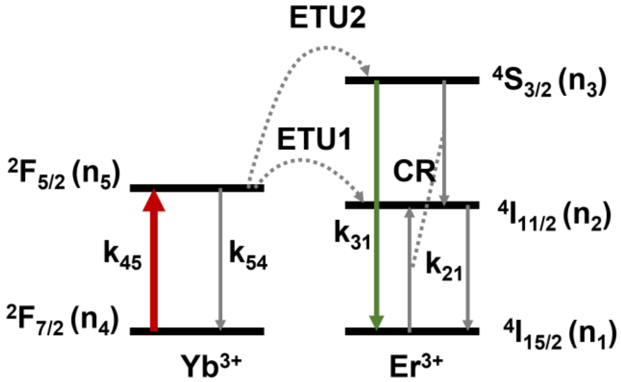

Figure S7. Schematic representation of the upconversion mechanism of the Er^{3+} green luminescence at 540 nm sensitized by Yb^{3+} under 980 nm excitation. The energy level diagram of Er^{3+} is simplified to include the most relevant states. The transitions and rates of interest are shown (red for excitation, green for emission, and dashed for energy transfer).

The kinetics of this upconversion system can be described by a set of differential equations:

$$\frac{dn_2}{dt} = -k_{21}n_2 + k_{52}n_1n_5 - k_{23}n_2n_5 + 2C_1n_1n_3 \qquad (1)$$

$$\frac{dn_3}{dt} = -k_{31}n_3 + k_{23}n_2n_5 - C_1n_1n_3 \qquad (2)$$

$$\frac{dn_5}{dt} = k_{45}n_4 - k_{54}n_5 - k_{52}n_1n_5 - k_{23}n_2n_5 \quad (3)$$

where n_i is the population density of energy level i, k_{ij} is the rate constant for the transition between levels i and j, k_{52} and k_{23} are the coefficients for ETU1 and ETU2, respectively, and C_1 the rate constant for the cross-relaxation process. We consider the dependence of the pumping process on the excitation photon flux P and assume that the population densities of states 1 and 4 are constant.

$$k'_{45} = P\sigma n_4 \quad (4)$$

$$k'_5 = k_{54} + k_{52}\, n_1 \quad (5)$$

$$k'_{52} = k_{52}\, n_1 \quad (6)$$

where σ is the absorption cross-section of Yb^{3+}. We also assume that $k_{23}n_2n_5$ is negligible in comparison to $k_{52}n_1n_5$ in Eq. (1) and Eq. (3), essentially representing a weak-excitation condition. Then, Eq. (3) transforms into Eq. (7).

$$\frac{dn_5}{dt} = k'_{45}u(t) - k'_5 n_5 \quad (7)$$

$$u(t) = \begin{cases} 1 & \text{for } t < t_p \\ 0 & \text{for } t_p < t < T \end{cases} \quad (8)$$

where *u(t)* is the normalized step-like excitation function.

The parameters for the simulations were set as stated in Table S2, to represent the typical range of those values for Yb^{3+}-Er^{3+}-codoped UCNPs. When fixed, the parameters were set to their typical values.

Table S2. Parameter values used in the simulations of the dependence of the UCL intensity on the excitation pulse width.

Parameter	Minimum	Typical	Maximum
Period (s)	0.006	-	60
Duty cycle (%)	0.05	0.2	3
Excitation intensity (W cm^{-2})	5	100	500
Decay time of the Yb^{3+} $^2F_{5/2}$ state (ms)	0.05	1	5
Decay time of the Er^{3+} $^4I_{11/2}$ state (ms)	0.1	1.32	10
Decay time of the Er^{3+} $^4S_{3/2}$ state (ms)	0.01	0.1	1
Energy transfer rate from Yb^{3+} $^2F_{5/2}$ to Er^{3+} $^4I_{15/2}$ (cm^3 s^{-1})	2×10^{-17}	2×10^{-16}	2×10^{-15}
Energy transfer rate from Yb^{3+} $^2F_{5/2}$ to Er^{3+} $^4I_{11/2}$ (cm^3 s$^{-1)}$	2×10^{-16}	2×10^{-15}	2×10^{-14}
Cross-relaxation rate (cm^3 s^{-1}) (see ref. [3])	0	0	10^{-16}

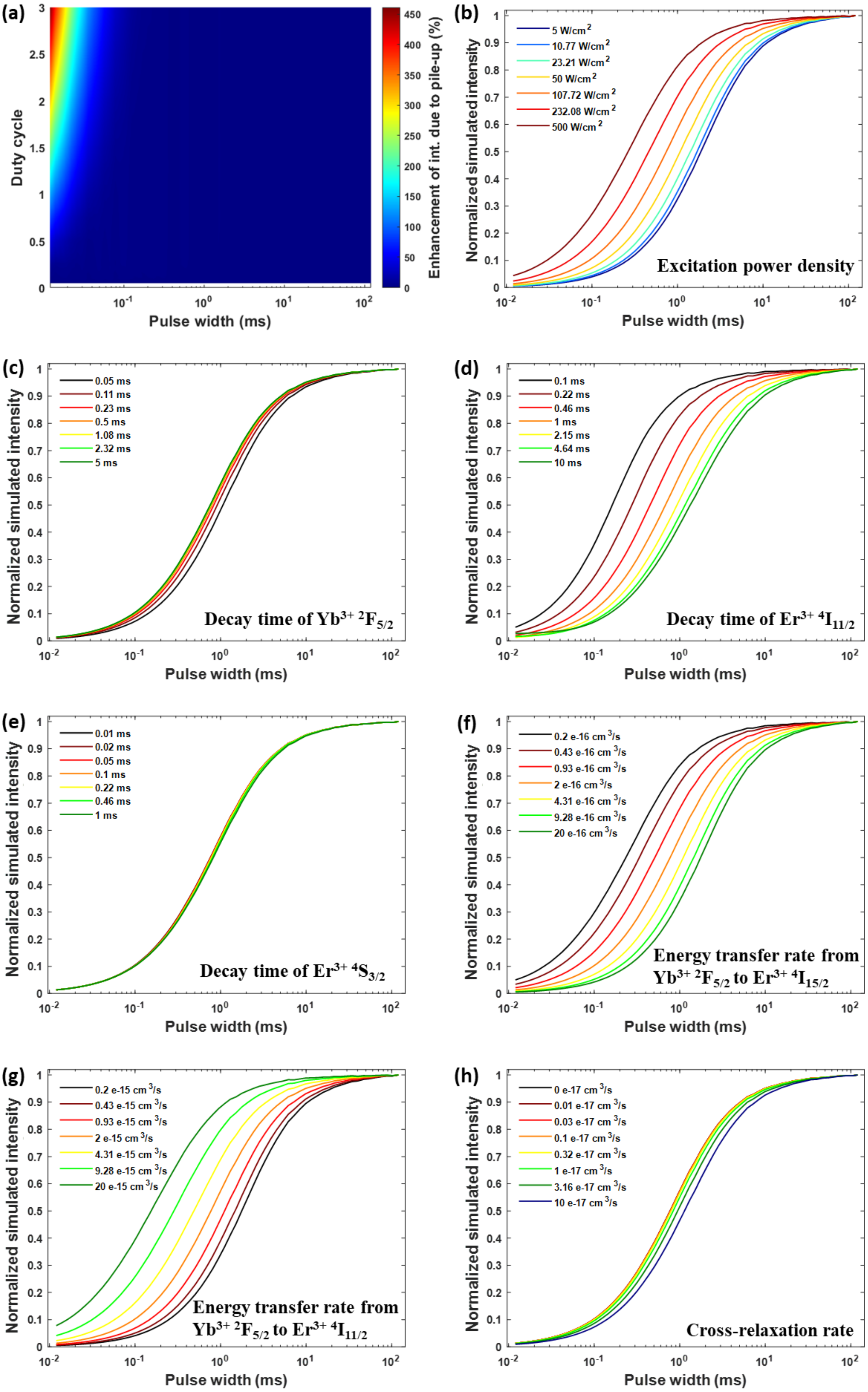
(a)
Duty cycle
Pulse width (ms)
Enhancement of int. due to pile-up (%)
(b)
5 W/cm^2
10.77 W/cm^2
23.21 W/cm^2
50 W/cm^2
107.72 W/cm^2
232.08 W/cm^2
500 W/cm^2
Excitation power density
Normalized simulated intensity
Pulse width (ms)
(c)
0.05 ms
0.11 ms
0.23 ms
0.5 ms
1.08 ms
2.32 ms
5 ms
Decay time of Yb^{3+} $^2F_{5/2}$
Normalized simulated intensity
Pulse width (ms)
(d)
0.1 ms
0.22 ms
0.46 ms
1 ms
2.15 ms
4.64 ms
10 ms
Decay time of Er^{3+} $^4I_{11/2}$
Normalized simulated intensity
Pulse width (ms)
(e)
0.01 ms
0.02 ms
0.05 ms
0.1 ms
0.22 ms
0.46 ms
1 ms
Decay time of Er^{3+} $^4S_{3/2}$
Normalized simulated intensity
Pulse width (ms)
(f)
0.2 e-16 cm^3/s
0.43 e-16 cm^3/s
0.93 e-16 cm^3/s
2 e-16 cm^3/s
4.31 e-16 cm^3/s
9.28 e-16 cm^3/s
20 e-16 cm^3/s
Energy transfer rate from Yb^{3+} $^2F_{5/2}$ to Er^{3+} $^4I_{15/2}$
Normalized simulated intensity
Pulse width (ms)
(g)
0.2 e-15 cm^3/s
0.43 e-15 cm^3/s
0.93 e-15 cm^3/s
2 e-15 cm^3/s
4.31 e-15 cm^3/s
9.28 e-15 cm^3/s
20 e-15 cm^3/s
Energy transfer rate from Yb^{3+} $^2F_{5/2}$ to Er^{3+} $^4I_{11/2}$
Normalized simulated intensity
Pulse width (ms)
(h)
0 e-17 cm^3/s
0.01 e-17 cm^3/s
0.03 e-17 cm^3/s
0.1 e-17 cm^3/s
0.32 e-17 cm^3/s
1 e-17 cm^3/s
3.16 e-17 cm^3/s
10 e-17 cm^3/s
Cross-relaxation rate
Normalized simulated intensity
Pulse width (ms)

Figure S8. Calculated results of the dependence of the intensity of a standard two-photon upconversion emission band on the excitation pulse width. Parameter values included in Table S2. Effects of **(a)** duty cycle, **(b)** the average excitation power density, **(c)** the lifetime of the excited state of the sensitizer ion, **(d)** the lifetime of the intermediate state of the activator ion, **(e)** the lifetime of two-photon upconversion luminescence emitting state of the activator ion, **(f)** the energy transfer rate of the first energy transfer step involved in the upconversion process, **(g)** the energy transfer rate of the second energy transfer step involved in the upconversion process, and **(h)** the cross-relaxation rate.

S6. Theoretical analysis of the excitation pulse duration response of two-photon upconversion luminescence

In this analysis, we ignore the cross-relaxation process. Then the rate equations can be described by:

$$\frac{dn_5}{dt} = k'_{45}u(t) - k'_5 n_5 \tag{7}$$

$$\frac{dn_2}{dt} = k'_{52}n_5 - k_{21}n_2 \tag{9}$$

$$\frac{dn_3}{dt} = k_{23}n_2n_5 - k_{31}n_3 \tag{10}$$

Applying Laplace transforms on both sides of Eq. (7):

$$sN_5(s) - n_5(0) = k'_{45}U(s) - k'_5N_5(s) \tag{11}$$

$$N_5(s) = \frac{k'_{45}}{(s + k'_5)}U(s) + \frac{n_5(0)}{s + k'_5} \tag{12}$$

Then, applying reverse Laplace transform and considering $n_5(0) = 0$:

$$n_5(t) = k'_{45}u(t) * e^{-k'_5 t} \tag{13}$$

If $t < t_p$, then:

$$n_{5_{t<t_p}}(t) = k'_{45}\int_0^t u(\tau)e^{-k'_5(t-\tau)}\,d\tau \tag{14}$$

$$n_{5_{t<t_p}}(t) = \frac{k'_{45}}{k'_5}\left(1 - e^{-k'_5 t}\right) \tag{15}$$

If $t_p < t < T$, considering Eq. (8):

$$n_{5_{t_p<t<T}}(t) = k'_{45}\int_0^{t_p} e^{-k'_5(t-\tau)}\,d\tau \tag{16}$$

$$n_{5_{t_p<t<T}}(t) = \frac{k'_{45}}{k'_5}e^{-k'_5 t}\left(e^{k'_5 t_p} - 1\right) \tag{17}$$

Applying Laplace transform on both sides of Eq. (9) yields:

$$sN_2(S) - n_2(0) = k'_{52}N_5(S) - k_{21}N_2(s) \tag{18}$$

$$N_2(s) = \frac{k'_{52}N_5(s)}{s + k_{21}} + \frac{n_2(0)}{s + k_{21}} \tag{19}$$

Then, applying reverse Laplace transform and assuming $n_2(0) = 0$ yields:

$$n_2(t) = k'_{52} n_5(t) * e^{-k_{21}t} \tag{20}$$

If $t < t_p$,

$$n_{2_{t<t_p}}(t) = k'_{52} \int_0^t n_5(\tau)_{t<t_p} e^{-k_{21}(t-\tau)}\, d\tau \tag{21}$$

$$n_{2_{t<t_p}}(t) = \frac{k'_{52}k'_{45}}{k'_5} e^{-k_{21}t} \int_0^t \left(1 - e^{-k'_5\tau}\right) e^{k_{21}\tau} d\tau \tag{22}$$

$$n_{2_{t<t_p}}(t) = \frac{k'_{52}k'_{45}}{k'_5}\left(\frac{1}{k_{21}} - \frac{1}{k_{21}-k'_5} e^{-k'_5 t} + \frac{k'_5}{(k_{21}-k'_5)k_{21}} e^{-k_{21}t}\right) \tag{23}$$

If $t_p < t < T$,

$$n_{2_{t_p<t<T}}(t) = k'_{52} \int_0^t n_5(\tau) e^{-k_{21}(t-\tau)}\, d\tau \tag{24}$$

$$n_{2_{t_p<t<T}}(t) = k'_{52}\left[\int_0^{t_p} n_{5_{t<t_p}}(\tau) e^{-k_{21}(t-\tau)}\, d\tau + \int_{t_p}^t n_{5_{t_p<t<T}}(\tau) e^{-k_{21}(t-\tau)}\, d\tau\right] \tag{25}$$

$$n_{2_{t_p<t<T}}(t) = \frac{k'_{52}k'_{45}}{k'_5(k_{21}-k'_5)}\left[\left(e^{k'_5 t_p} - 1\right) e^{-k'_5 t} - \frac{k'_5}{k_{21}}\left(e^{k_{21}t_p} - 1\right) e^{-k_{21}t}\right] \tag{26}$$

Then applying Laplace transform on both sides of Eq. (10) yields:

$$sN_3(s) - n_3(0) = k_{23}\mathcal{L}\{n_2 n_5\} - k_{31}N_3(s) \tag{27}$$

$$N_3(s) = \frac{n_3(0)}{s + k_{31}} + \frac{k_{23}\mathcal{L}\{n_2 n_5\}}{s + k_{31}} \tag{28}$$

Then, applying reverse Laplace transform and assuming $n_2(0) = 0$ yields:

$$n_3(t) = k_{23}(n_2 n_5) * e^{-k_{31}t} \tag{29}$$

If $t < t_p$

$$n_{3_{t<t_p}}(t) = k_{23} \int_0^t n_{2_{t<t_p}}(\tau) n_{5_{t<t_p}}(\tau) e^{-k_{31}(t-\tau)} d\tau \tag{30}$$

Then it can be obtained that

$$n_{3_{t<t_p}}(t) = \frac{k_{23}k'_{52}k'^2_{45}}{k'^2_5}\Bigg[\frac{1}{k_{21}k_{31}} + \left(-\frac{1}{k_{21}k_{31}} + \frac{1}{(k_{21}-k'_5)(k_{31}-k'_5)} - \frac{k'_5}{(k_{21}-k'_5)(k_{31}-k_{21})k_{21}} + \frac{1}{(k_{31}-k'_5)k_{21}} - \frac{1}{(k_{21}-k'_5)(k_{31}-2k'_5)} + \frac{k'_5}{(k_{21}-k'_5)(k_{31}-k'_5-k_{21})k_{21}}\right) e^{-k_{31}t} + \left(-\frac{1}{(k_{31}-k'_5)k_{21}} - \frac{1}{(k_{21}-k'_5)(k_{31}-k'_5)}\right) e^{-k'_5 t} + \frac{k'_5}{(k_{21}-k'_5)(k_{31}-k_{21})k_{21}} e^{-k_{21}t} + \frac{1}{(k_{21}-k'_5)(k_{31}-2k'_5)} e^{-2k'_5 t} + \left(-\frac{k'_5}{(k_{21}-k'_5)(k_{31}-k'_5-k_{21})k_{21}}\right) e^{(-k'_5-k_{21})t}\Bigg] \tag{31}$$

or

$$n_{3_{t<t_p}}(t) = A\left[B + Ce^{-k_{31}t} + De^{-k'_5 t} + Ee^{-k_{21}t} + Fe^{-2k'_5 t} + Ge^{(-k'_5-k_{21})t}\right] \tag{32}$$

with

$$A = \frac{k_{23}k'_{52}k'^2_{45}}{k'^2_5} \tag{33}$$

$$B = \frac{1}{k_{31}k_{21}} \tag{34}$$

$$C = -(B + D + E + F + G) \tag{35}$$

$$D = -\frac{1}{(k_{21} - k'_5)(k_{31} - k'_5)} - \frac{1}{k_{21}(k_{31} - k'_5)} \tag{36}$$

$$\mathrm{E} = \frac{k'_5}{(k_{21} - k'_5)(k_{31} - k_{21})k_{21}} \tag{37}$$

$$F = \frac{1}{(k_{21} - k'_5)(k_{31} - 2k'_5)} \tag{38}$$

$$G = -\frac{k'_5}{(k_{21} - k'_5)(k_{31} - k'_5 - k_{21})k_{21}} \tag{39}$$

If $t_p < t < T$,

$$n_{3_{t_p<t<T}}(t) = k_{23}\left[\int_0^{t_p} n_{2_{t<t_p}}(\tau)n_{5_{t<t_p}}(\tau)e^{-k_{31}(t-\tau)}d\tau + \int_{t_p}^{t} n_{2_{t_p<t<T}}(\tau)n_{5_{t_p<t<T}}(\tau)e^{-k_{31}(t-\tau)}d\tau\right] \tag{40}$$

$$n_{3_{t_p<t<T}}(t) = A\left[\left[C + (B - F - G)e^{k_{31}t_p} + (D + 2F + G)e^{(k_{31}-k'_5)t_p} + (E + G)e^{(k_{31}-k_{21})t_p}\right]e^{-k_{31}t} + F\left(e^{k'_5t_p} - 1\right)^2 e^{-2k'_5t} + G\left(e^{k'_5t_p} - 1\right)\left(e^{k_{21}t_p} - 1\right)e^{-(k_{21}+k'_5)t}\right] \tag{41}$$

The average emitted intensity per pulse of in a period T in the excitation pulse-duration response is:

$$F(t_p) = \frac{1}{t_p}\int_0^T n_3(t)dt \tag{42}$$

i.e.

$$F(t_p) = \frac{1}{t_p}\left[\int_0^{t_p} n_{3_{t<t_p}}(t)dt + \int_{t_p}^{T} n_{3_{t_p<t<T}}(t)dt\right] \tag{43}$$

Given the period T is sufficiently long that allow the excited state populations to completely relax to the ground state, i.e. $e^{-k_iT} \approx 0$, it yields

$$F(t_p) = \frac{A}{t_p}\left(Bt_p + H\left(e^{-k_{21}t_p} - 1\right) + I\left(e^{-k'_5t_p} - 1\right)\right) \tag{44}$$

$$H = -\frac{{k'_5}^2}{{k_{21}}^2k_{31}\left({k_{21}}^2 - {k'_5}^2\right)} \tag{45}$$

$$I = \frac{k_{21}}{k_{31}k'_5\left({k_{21}}^2 - {k'_5}^2\right)} \quad (46)$$

Noting that $B = \frac{1}{k_{31}k_{21}}$, Eq. (44) indicates the shape of normalized $F(t_p)$ doesn't show dependence on k_{31}. In addition, typically $k'_5 \gg k_{21}$, so the shape of normalized $F(t_p)$ is dominantly affected by the rate constant k_{21}.

S7. Effect of excitation intensity

The variation of the excitation power can alter the kinetics of UCNPs, mainly allowing to populate higher energy levels. The dependence of the emitted intensity on the excitation pulse width was assessed at ten different values of average excitation power (Figure S9a-c and Figure 2a) for different emission bands of Yb^{3+}-Er^{3+}-codoped UCNPs. The effect of the excitation intensity was also simulated for the emission band at around 540 nm, reproducing the tendency of the experimental results for that emission band (Figure S8b, see details of the method in SI Section S5). A replot of the dependence of the emitted intensity on the excitation pulse width at ten different values of average excitation power allows to estimate the variation of the dependence of the emission intensity on the excitation average power for different excitation pulse widths and continuous wave excitation conditions (Figure S9d-f and Figure 2b) and to extract from those data the parameter *n* (the slope in Figure S9d-f), usually related to the number of photons involved in an upconversion process (Figure S10).

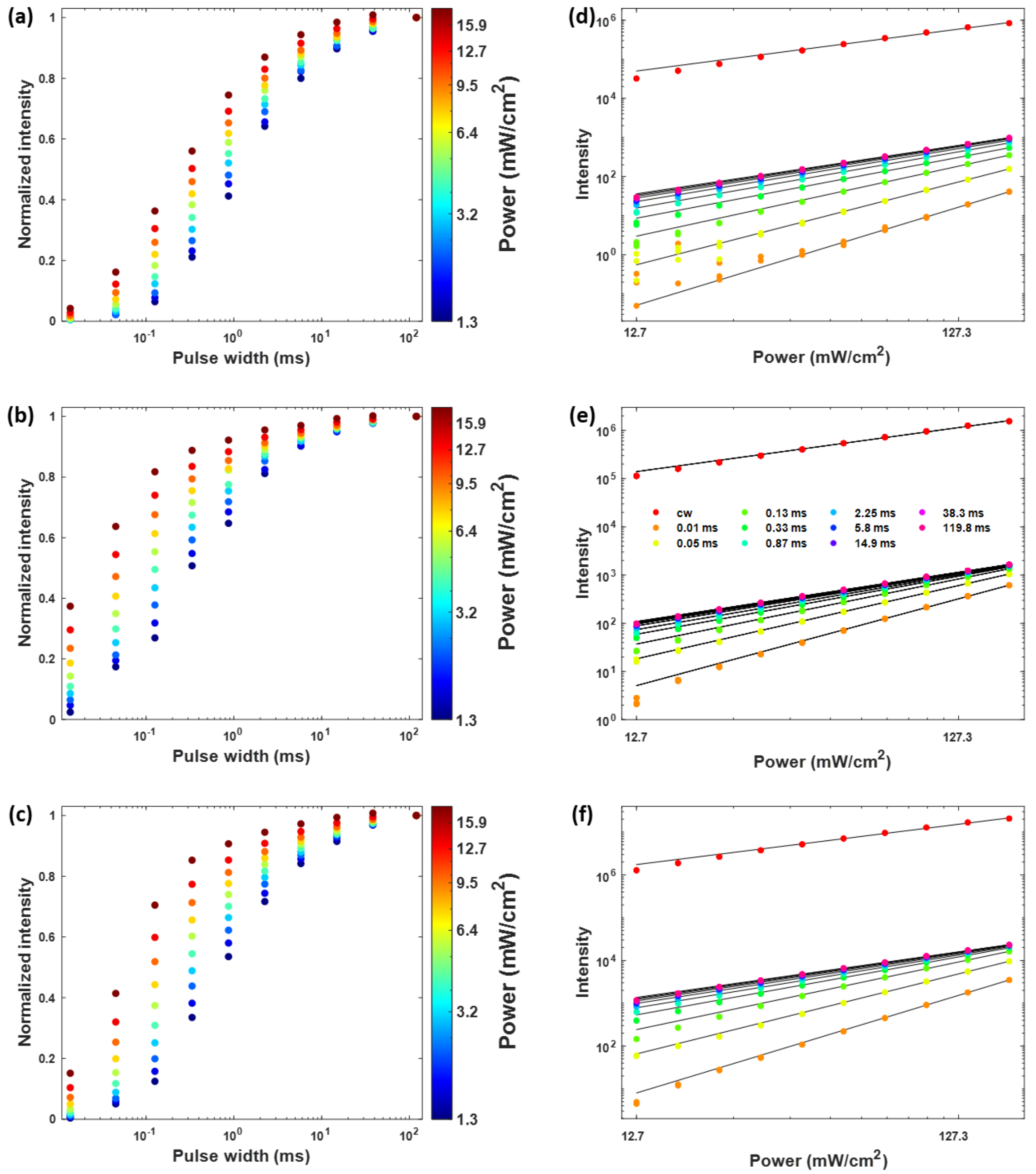

Figure S9. Dependence of the emission intensity of the **(a)** blue, **(b)** green (at 520 nm), and **(c)** red emission bands of Yb^{3+}-Er^{3+}-codoped UCNPs with slow kinetics on the excitation pulse width at different average excitation powers of the modulated 980 nm laser (duty cycle 0.2%). Each data point is the average of three measurements. Standard deviation is much smaller than the symbol size. Intensity of the Yb^{3+}-Er^{3+}-codoped UCNPs with slow kinetics as a function of average excitation power at 980 nm modulated at different pulse widths (duty cycle 0.2%) and continuous wave for the **(d)** blue, **(e)** the green (at 520 nm), and **(f)** red emission bands. Each data point is the average of three measurements. Standard deviation is much smaller than the symbol size. Black lines are fits to $\langle UCL(\lambda, w)\rangle \propto (I_{\mathrm{exc}})^n$. Average value of R^2=0.9994 ± 0.0012.

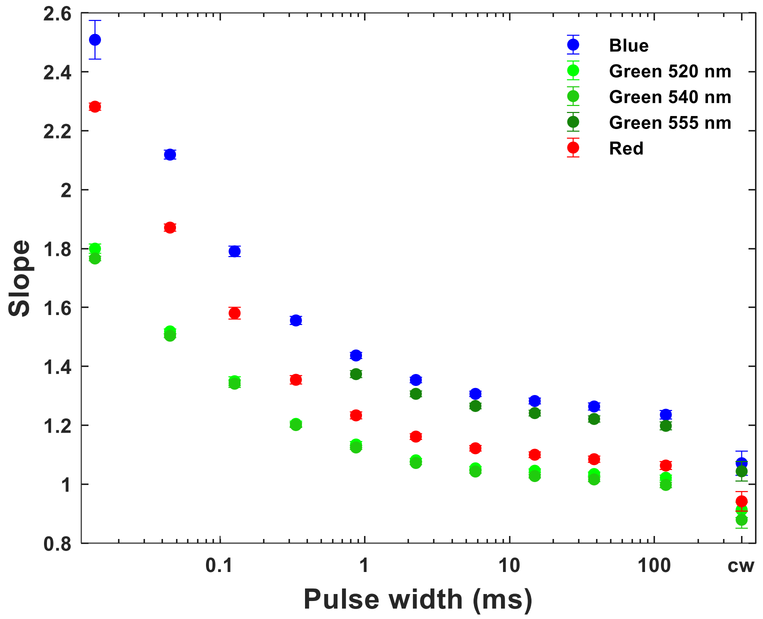

Figure S10. Variation of the slope (parameter *n*) from the fitting of the emission intensity as a function of average excitation power (Figure 2b and S9d-f) for the emission bands of the Yb^{3+}-Er^{3+}-codoped UCNPs with slow kinetics as a function of average excitation power at 980 nm modulated at different pulse widths (duty cycle 0.2%) or continuous-wave excitation. Average value of R^2=0.9995 ± 0.0012. Error bars obtained from 95% confidence interval of *n* from the fitting to $\langle UCL(\lambda, w)\rangle > \propto (I_{\rm exc})^n$.

S8. Effect of temperature

The variation of the temperature affects the kinetics of UCNPs, varying the nonradiative decay rate. The dependence of the emitted intensity on the excitation pulse width was assessed at nine different values of temperature (Figure S11a-d and Figure 4c) for different emission bands of Yb^{3+}-Er^{3+}-codoped UCNPs. A replot of the dependence of the emitted intensity on the excitation pulse width at nine different values of temperature allows to estimate the variation of the dependence of the emission intensity on temperature for different excitation pulse widths and continuous wave excitation conditions (Figure S11e-f and Figure 4d).

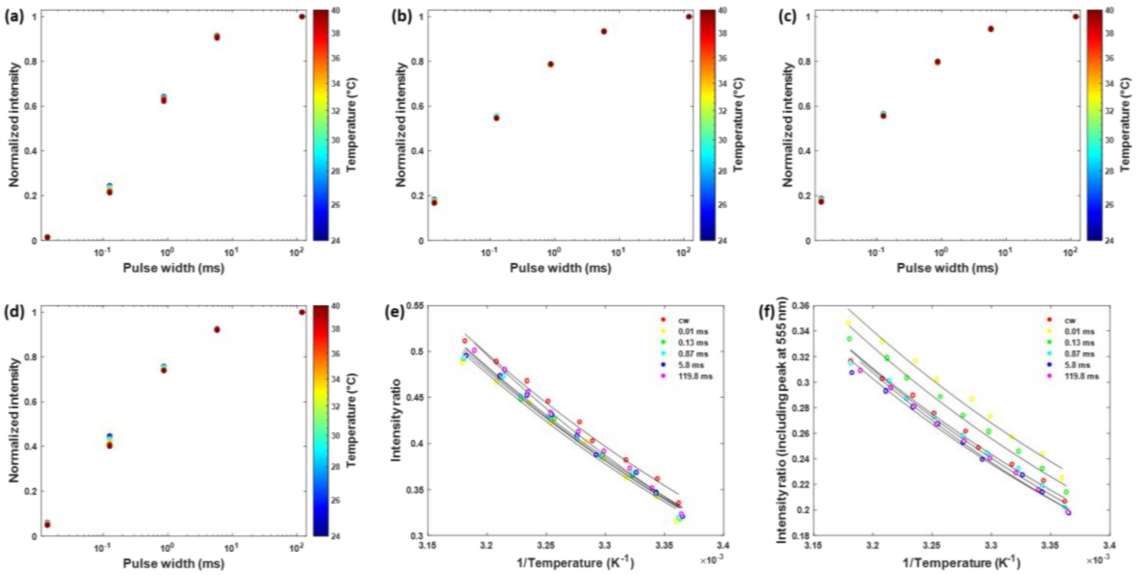

Figure S11. Dependence of the intensity of the **(a)** blue, **(b)** green at 520 nm, **(c)** green at 540 nm, and **(d)** red emission bands of Yb^{3+}-Er^{3+}-codoped UCNPs on the excitation pulse width of the 980 nm modulated laser (duty cycle 0.2%) at different temperatures. Each data point is the average of three measurements. Standard deviation is much smaller than the symbol size. Temperature calibration of the ratio between the intensity of the green bands of Yb^{3+}-Er^{3+}-codoped UCNPs with slow kinetics obtained under different excitation modulation conditions (with constant average excitation power density of 56 mW cm^{-2}) **(e)** excluding and **(f)** considering the spectrally overlapping emission at around 555 nm. Black lines are the fits to $I_{520}/I_{540} \propto \exp(-\Delta E/k_B T)$, where k_B is the Boltzmann constant, ΔE is the energy mismatch between the involved emitting energy levels, and T is the temperature in Kelvin. Average value of $R^2 = 0.987 \pm 0.004$. Each data point is the average of three measurements. Standard deviation is much smaller than the symbol size.

S9. Er^{3+} emission green bands ratio

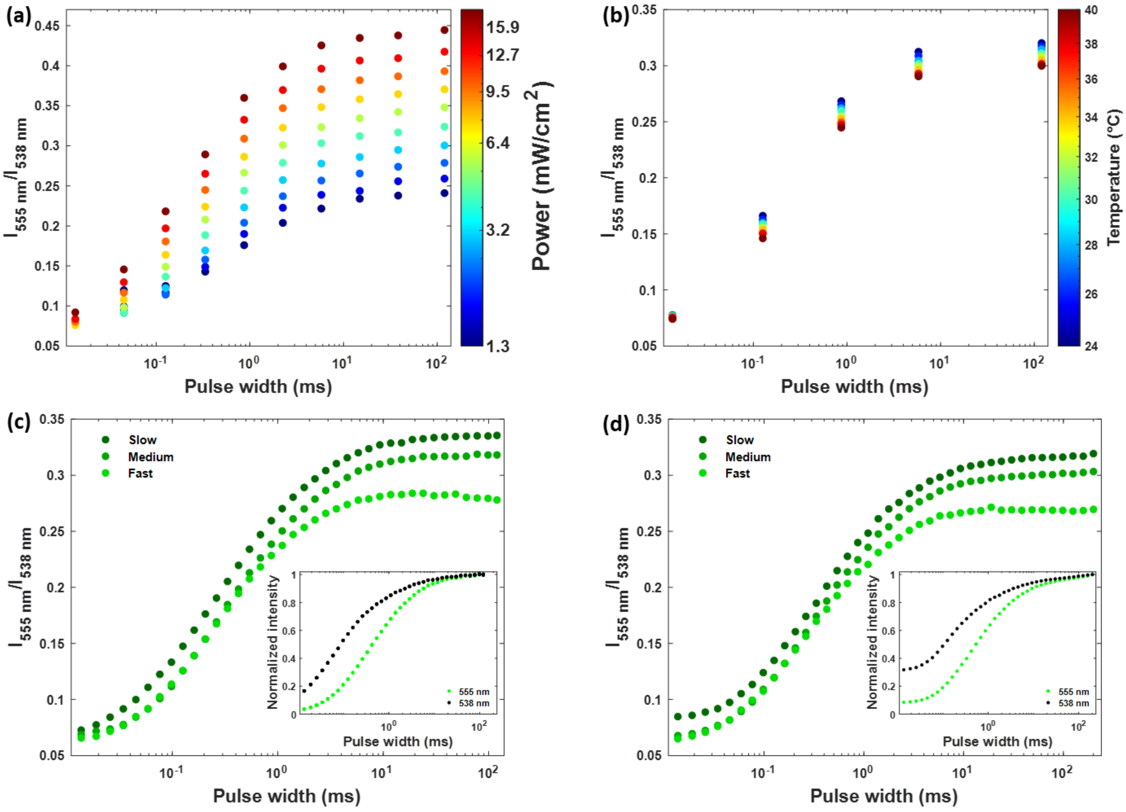

Figure S12. Dependence of the intensity ratio of the peak coming from the $^2H_{9/2} \rightarrow {}^4I_{13/2}$ transition (555 nm) and the emission coming from the $^4S_{3/2} \rightarrow {}^4I_{15/2}$ transition (540 nm) for the Yb^{3+}-Er^{3+}-codoped UCNPs with slow kinetics on the excitation pulse width (duty cycle 0.2%) **(a)** at different average excitation powers of the modulated 980 nm laser (room temperature) and **(b)** at different temperatures (56 mW cm^{-2} average excitation power density). Each data point is the average of three measurements. Standard deviation is much smaller than the symbol size. Dependence of the intensity ratio of the peak coming from the $^2H_{9/2} \rightarrow {}^4I_{13/2}$ transition (555 nm) and the emission coming from the $^4S_{3/2} \rightarrow {}^4I_{15/2}$ transition (540 nm) for the Yb^{3+}-Er^{3+}-codoped UCNPs with different kinetics (Figure S2) on the excitation pulse width of the modulated 980 nm laser at duty cycle **(c)** 0.2% and **(d)** 2%. Insets show the dependence of the intensities of the emission bands at 540 and 555 nm of the Yb^{3+}-Er^{3+}-codoped UCNPs with slow kinetics on the excitation pulse width of the modulated 980 nm laser with the respective duty cycle value. Measured at room temperature and 56 mW cm^{-2} average excitation power density. Each data point is the average of three measurements. Standard deviation is much smaller than the symbol size.

S10. Application to Yb^{3+}-Tm^{3+}-codoped nanoparticles

The method developed in this work was applied to a different type of UCNPs: $NaYF_4$:20%Yb,0.5%Tm (see synthesis procedure in SI section S1). These UCNPs show a diameter of 28.1 ± 1.5 nm (see Figure S13a-b). The emission bands identified in Figures S13c-d had their kinetics characterized (Figure S13e-h) and the maximum possible duty cycle assessed (Figure S13i). The dependence of these emission band intensities on the excitation pulse width was obtained, showing that several of them originated from the same energy levels (showing the same dependence on the variation of the excitation pulse width) (Figure S13j).

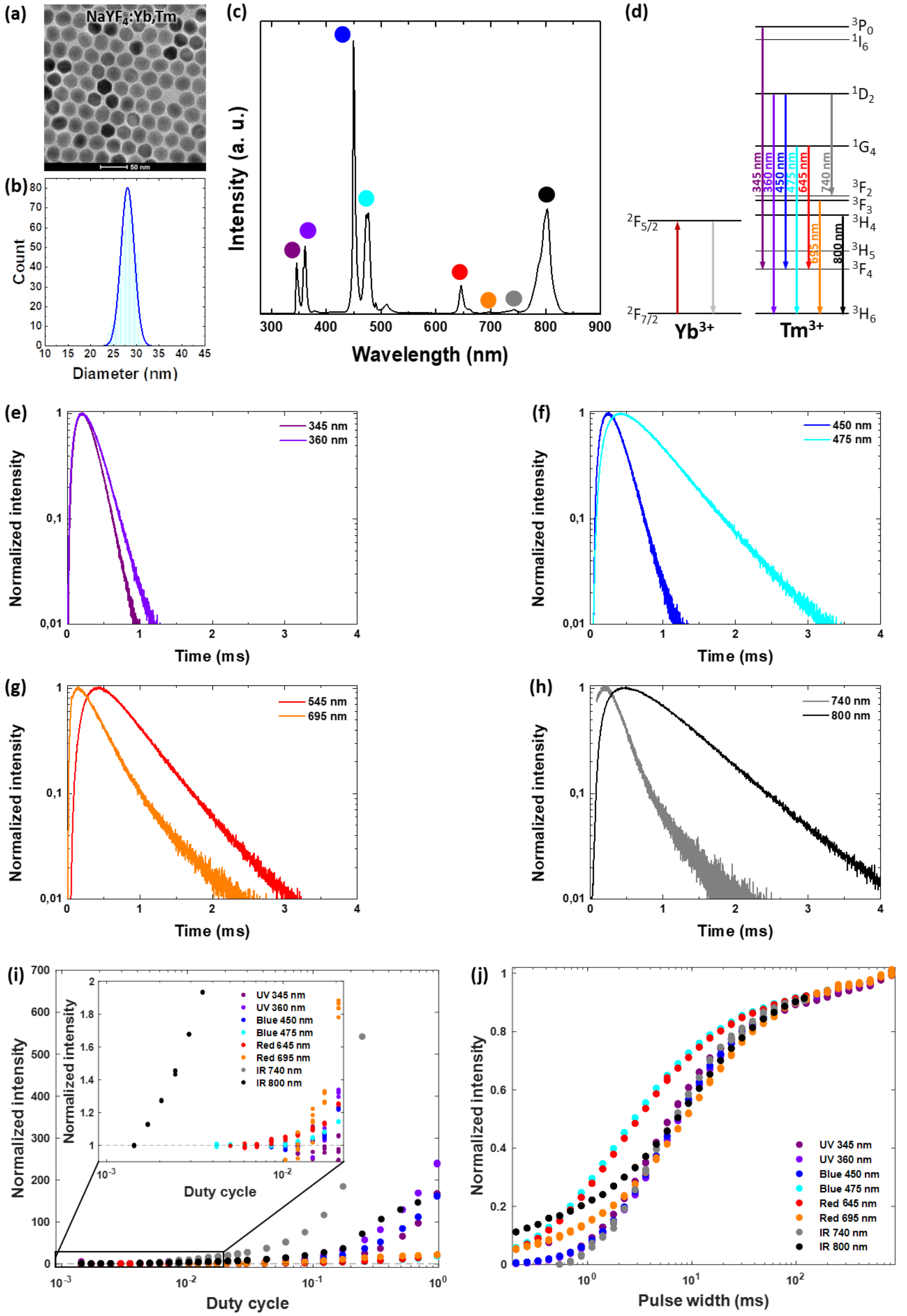

Figure S13. **(a)** TEM image (scale bar 50 nm) and **(b)** size histogram (obtained from the analysis of several TEM images) of $NaYF_4$:Yb,Tm nanoparticles. **(c)** Emission spectrum of $NaYF_4$:Yb,Tm nanoparticles under excitation at 980 nm. Dots show the color codes to identify data related to the different emission bands. **(d)** Schematic energy levels diagram for Yb^{3+} and Tm^{3+} ion showing the transitions of interest. **(e)-(h)** Intensity decay for the different emission bands of Tm^{3+} ion

under pulsed excitation at 980 nm. **(i)** Dependence of the emission intensity of different emission bands of the Yb^{3+}-Tm^{3+}-codoped UCNPs under modulated 980 nm excitation (0.02 ms pulse width) on the excitation modulation duty cycle. Inset is a zoom of the region with lower duty cycle value. **(j)** Dependence of the intensities of different emission bands of the Yb^{3+}-Tm^{3+}-codoped UCNPs on the excitation pulse width of the modulated 980 nm laser (duty cycle 2% for UV and visible emission bands and duty cycle 0.2% for IR emission bands). Each data point is the average of three measurements. Standard deviation is much smaller than the symbol size.

References

(1) Liu, Q.; Liu, H.; Li, D.; Qiao, W.; Chen, G.; Ågren, H. Microlens array enhanced upconversion luminescence at low excitation irradiance. *Nanoscale* **2019**, *11* (29), 14070-14078.

(2) Kostiv, U.; Engstová, H.; Krajnik, B.; Šlouf, M.; Proks, V.; Podhorodecki, A.; Ježek, P.; Horák, D. Monodisperse core-shell NaYF4: Yb3+/Er3+@ NaYF4: Nd3+-PEG-GGGRGDSGGGY-NH2 nanoparticles excitable at 808 and 980 nm: Design, surface engineering, and application in life sciences. *Frontiers in chemistry* **2020**, *8*, 497.

(3) Bergstrand, J.; Liu, Q.; Huang, B.; Peng, X.; Würth, C.; Resch-Genger, U.; Zhan, Q.; Widengren, J.; Ågren, H.; Liu, H. On the decay time of upconversion luminescence. *Nanoscale* **2019**, *11* (11), 4959-4969.
